# *Hevea brasiliensis* coniferaldehyde-5-hydroxylase (*HbCAld5H*) regulates xylogenesis, structure and lignin chemistry of xylem cell wall in *Nicotiana tabacum*

**DOI:** 10.1007/s00299-020-02619-8

**Published:** 2020-10-17

**Authors:** S. Pramod, Thakurdas Saha, K. Rekha, P. B. Kavi Kishor

**Affiliations:** 1 Advanced Centre for Molecular Biology and Biotechnology, Rubber Research Institute of India, Rubber Board, Kottayam, Kerala 686009 India; 2 Department of Biotechnology, Vignan’s Foundation for Science, Technology & Research, Vadlamudi, Guntur, 522213 Andhra Pradesh India; 3 Present Address: Department of Forest Genetics and Plant Physiology, Umea Plant Science Centre, Swedish University of Agricultural Sciences, 901-87 Umea, Sweden

**Keywords:** Coniferaldehyde-5-hydroxylase, *Hevea brasiliensis*, Xylogenesis, Cell wall structure, Lignin, S/G ratio, Xylans

## Abstract

**Key message:**

**The HbCAld5H1 gene cloned from Hevea brasiliensis regulates the cambial activity, xylem differentiation, syringyl–guaiacyl ratio, secondary wall structure, lignification pattern and xylan distribution in xylem fibres of transgenic tobacco plants.**

**Abstract:**

Molecular characterization of lignin biosynthesis gene coniferaldehyde-5-hydroxylase (*CAld5H)* from *Hevea brasiliensis* and its functional validation was performed. Both sense and antisense constructs of *HbCAld5H1* gene were introduced into tobacco through *Agrobacterium*-mediated genetic transformation for over expression and down-regulation of this key enzyme to understand its role affecting structural and cell wall chemistry. The anatomical studies of transgenic tobacco plants revealed the increase of cambial activity leading to xylogenesis in sense lines and considerable reduction in antisense lines. The ultra-structural studies showed that the thickness of secondary wall (S2 layer) of fibre had been decreased with non-homogenous lignin distribution in antisense lines, while sense lines showed an increase in S2 layer thickness. Maule color reaction revealed that syringyl lignin distribution in the xylem elements was increased in sense and decreased in antisense lines. The immunoelectron microscopy revealed a reduction in LM 10 and LM 11 labelling in the secondary wall of antisense tobacco lines. Biochemical studies showed a radical increase in syringyl lignin in sense lines without any significant change in total lignin content, while S/G ratio decreased considerably in antisense lines. Our results suggest that *CAld5H* gene plays an important role in xylogenesis stages such as cambial cell division, secondary wall thickness, xylan and syringyl lignin distribution in tobacco. Therefore, *CAld5H* gene could be considered as a promising target for lignin modification essential for timber quality improvement in rubber.

**Electronic supplementary material:**

The online version of this article (10.1007/s00299-020-02619-8) contains supplementary material, which is available to authorized users.

## Introduction

Lignin is an important biopolymer, which imparts structural and mechanical support to plant cell wall. The properties such as rigidity and hydrophobicity of the cell wall have been attributed to lignin content, which facilitates water transport through vascular tissues and mechanical defence to the plant against pathogens (Vanholme et al. 2008; Li and Chapple 2010). Lignin also represents the second most abundant biopolymer on earth after cellulose, which constitutes about 30% of the total biomass produced in the biosphere and form the major form of carbon sink due to its resistance to biodegradation (Boudet et al. 2003; Carocha et al. 2015). Being an integral part of xylem cell wall, lignin plays a crucial role in growth, development and survival of terrestrial vascular plants by providing mechanical rigidity and hydraulic conductivity under various biotic and abiotic stress conditions. The biosynthesis of lignin involves the oxidative polymerization of three hydroxycinnamyl alcohol precursors namely p-coumaryl, coniferyl and sinapyl alcohols and its composition varies between different plant species. The dicotyledonous plant has mainly guaiacyl and syringyl lignin units derived from coniferyl and sinapyl alcohols, respectively, and differ in their degree of hydroxylation and methoxylation in their aromatic ring (Boerjan et al. 2003). This result in different types of intermonomeric linkages between monomers within the lignin polymers and the lignin monomeric composition is likely to be the key element of several properties of lignin and cell wall (Ralph et al. 2019; Gui et al. 2020). The composition of lignin monomeric units has a critical role in wood properties and due to the resistance to chemical degradation by highly condensed guaiacyl units, lignin is a major hurdle in industrial processing of wood biomass for paper and pulp manufacturing, bioethanol production etc. (Hallac et al. 2010; Li et al. 2010; Garcia et al. 2014). Therefore, understanding the genetic regulation of syringyl:guaiacyl (S/G) ratio for exploring the potential of genetic engineering to tailor lignin monomeric composition has been a major focus of research in tree biotechnology.

Several genes involved in lignin biosynthetic pathway such as cinnamate-4-ligase (*4CL*), cinnamaldehyde dehydrogenase (*CAD*), cinnamoyl CoA reductase (*CCR*), coumarate-*O*-methyl transferase (*COMT*), ferulate-5-hydroxylase (*F-5-H*) have been studied extensively over last 2 decades and found effective in altering lignin content and monomeric composition in many plant species (Vanholme et al. 2008). Recent research revealed that apart from these genes which function in the upstream of lignin biosynthetic pathway, coniferaldehyde-5-hydroxylase (*CAld5H*) plays a major role in diverting the immediate precursor of guaiacyl lignin, coniferaldehyde into sinapaldehyde, the precursors of syringyl lignin pathway through a 5-hydroxylataion followed by 5-methoxylation coffeoate-O-methyl transferase (*COMT*) (Osakabe et al. 1999). These authors also reported that in the absence of coniferaldehyde, *CAld5H* can catalyse conversion of ferulate into 5-hydroxyferulate suggesting the potential role of *CAld5H* gene in ferulate metabolism also. Garcia et al. (2014) reported the restoration of syringyl lignin in the *Arabidopsis fah1-2* mutants transformed with *CAld5H*. The co-transformation experiments proved that *CAld5H* and *COMT* are the most efficient enzymes in regulating S/G ratio in higher plants as they function in the downstream of lignin specific pathway and their reaction products are the most likely to enter monolignol pathway (Chiang 2006). The *CAld5H* gene from *Eucalyptus* and rice are also demonstrated to be very efficient in increasing syringyl monomer ratio in lignins (Garcia et al. 2014; Takeda 2017). Therefore, *CAld5H* could be the most efficient target gene in tree biotechnology research for engineering plant cell wall chemistry for future needs.

In the present study, we characterized *CAld5H* genes from *Hevea brasiliensis*, a tropical deciduous tree species, used as a primary source of natural rubber but it also bears immense timber value for various industrial applications. We isolated two isoforms of this gene and designated as *HbCAld5H1* and *HbCAld5H2*. After examining the expression levels in various tissues, the *HbCAld5H1* was used as a candidate gene for molecular cloning and transformation into *Nicotiana tabacum* to assess the impact of down-regulation and overexpression of the gene on xylogenesis, structure and lignin chemistry.

## Materials and methods

### Cloning and transformation of *N. tabacum*

The *CAld5H* gene from *Hevea brasiliensis* (Clone RRII 105) was amplified from the genomic DNA as well as cDNA derived from young stem (second internode) total RNA, using gene specific primers (details of the primers listed in Table S1). The amplified genes were cloned into pGEM-T Easy Vector (Promega USA) for sequence characterization of recombinant plasmids. For plant transformation experiments, the sense and antisense constructs of *HbCAld5H1* gene were made by cloning the gene fragments into intermediate vector pRT101 and complete cassette into pCAMBIA1301 (Fig. S1). The sense construct consists of full length (1.542 kb) CAld5H cDNA. Two antisense constructs CAS-A and CAS-B having sizes of 842 bp and 473 bp, respectively, are the reverse sequences of nucleotides 156 to 628 (CAS-B) and 696 to 1538 (CAS-A) in CDS regions of full length *HbCAld5H1* gene. The plasmids were introduced into *Agrobacterium tumefaciens* (LBA4404). The details of the sequence used for antisense construct are provided in the supplementary method 1. Leaf disc derived from 8-week-old *Nicotiana tabacum* plants were transformed by *Agrobacterium* carrying pCAMBIA1301-sense and antisense *HbCAld5H1* constructs. Untransformed wild type (WT) tobacco plants served as controls. ß-glucuronidase (GUS) analysis of transformants and their hygromycin (50 mg/l) resistance were used as selective agents during in vitro plant regeneration. Transformed plants were grown in vitro for 6 weeks and then transferred to soil and grown to maturity in the green house. *T*_0_ transformants were allowed to self-pollinate to obtain homozygous lines. *T*_*1*_ seeds were harvested and subjected to selection on germination medium containing hygromycin.

### Molecular characterization of transgenic plants

Ten GUS-positive independent transformants were regenerated with *HbCAld5H1* sense construct, while 25-independent transformants were regenerated with *HbCAld5H1* antisense construct. Of these, three independent transformants (3-month-old) from each *HbCAld5H1* sense and antisense constructs (CAS-A and CAS-B) were selected along with wild-type (WT) plants and analysed by PCR and southern blotting. The *HbCAld5H1* gene specific primers were used for PCR confirmation of transgenic tobacco plants with *HbCAld5H1* sense and antisense transgenes. For southern blotting, genomic DNA was digested with *Sac*I, which had no cutting site in the transgene *HbCALd5H1*, separated by agarose gel electrophoresis and blotted onto nylon membranes (Hybond N, Amersham Biosciences, UK) using standard protocols (Sambrook and Russell 2001). DNA amplified with gene specific primers from pCAMBIA-sense and antisense *HbCAld5H1* (CAS-A and CAS-B) plasmids were used as probes after labelling with DIG DNA Labelling and Detection System (Boehringer Mannheim GmbH, Germany). The visualization of the probe-target hybrid was achieved by a chemiluminiscent assay using the DIG luminescent detection kit protocol (Roche, Diagnostics GmbH, Germany).

Expression of *CAld5H* transgene was quantified by qPCR. Total cDNA was used as the template for q-RT-PCR. The cDNA was prepared from the total RNA extracted from the leaves of WT and transgenic tobacco plants according to the manufacturer’s protocol of Ambion cDNA Kit (Thermo Scientific, USA). The qRT-PCR results were measured using 7500 SDS software (Applied Biosystems, USA). Tobacco β-actin, considered to be one of the most suitable reference genes in terms of stability for real-time PCR experiments (Bustin 2002; Schmidt and Delaney 2010) has been used as a reliable reference gene to find out the expression levels of *CAld5H* gene in transgenic tobacco (Chen et al. 2019). Therefore, the expression levels of tobacco β-actin gene as the reference gene with actin specific primers were quantified in parallel with the target genes as the internal control. Three biological samples and triplicate technical qRT-PCR reactions for each combination of primers and samples were analysed. Expression fold-change was calculated as 2^−∆∆Ct^ for each replicate.

### Light microscopy

For general histology, transverse sections (1–2 µm thick) taken with a diamond knife from the LR White embedded samples using an ultra-microtome (Leica UMO7, Germany) were stained with 0.05% toluidine blue O (Berlyn and Mikshe 1976). Sections were examined and photographed using Leica microscope (DM200) with a Canon digital camera (DM 150). For histochemical analysis, hand sections taken from stem and leaf tissues of control and transgenic lines were stained with toluidine blue O for general histology, phloroglucinol-HCl for lignin localization (Speer 1987) and Maule’s reaction (Meshitsuka and Nakano 1979) for syringyl lignin localization. Stained sections were observed and photographed using Leica DM200 microscope (Germany). Stem samples from control, lignin up- and down-regulated plants were macerated to measure the length and width of fibres and vessel elements. Small matchstick size stem pieces were macerated by incubating in Jeffrey’s fluid (Berlyn and Miksche 1976). After thorough washing in water, the macerated elements were stained with safranin O (Sigma, S-2255) before mounting in 50% glycerol. The length and width of fibres and vessel elements were measured with an ocular micrometer scale mounted in a research microscope. For each parameter, 100 readings were taken from randomly selected elements and they were statistically analyzed to determine the mean values.

### Transmission electron microscopy

Suitably trimmed (2 × 5 mm size) secondary xylem tissues were fixed in a mixture of 0.1% glutaraldehyde and 4% paraformaldehyde in 50 mM sodium cacodylate buffer for 4 h at room temperature. After washing in buffer, tissues were dehydrated in graded series of ethanol (30–95%, 15 min each, pure ethanol × 3, each for 20 min) and embedded in LR white resin as described elsewhere (Pramod et al. 2019). Transverse ultrathin sections having 70–80 nm thickness were prepared from the LR white embedded blocks using an ultra-microtome (Ultracut E, Leica, Germany) with a diamond knife and mounted on nickel grids. Sections were stained with 0.1% KMnO_4_ in citrate buffer for 45 min at room temperatures for lignin (Donaldson 1992). For immunolabelling, ultrathin sections of 90 nm thickness were taken from the LR white embedded blocks mounted on nickel grids. After suspension of the grids in buffer ‘A’ (composition: Tris-buffered saline containing 1% bovine serum albumin and 0.1% NaN_3_, pH 8.2) for 30 min at room temperature, the grids were incubated with LM10 or LM11 antibodies (1:20 dilution in buffer A) for 2 days at 4 ˚C. The labelling method was same for xyloglucan except the grids were incubated with goat anti-mouse secondary antibody labelled with 5 nm colloidal gold particle (BB International, UK). After three washings with buffer A for 15 min each, the grids were incubated with goat anti-rat secondary antibody labelled with 10 nm colloidal gold particles (BB International, UK) for 2 h at room temperature for the LM10 antibody (1:20 dilution in buffer ‘A’). For the control, some sections were also incubated only with secondary antibody. Finally, the grids were washed in six changes of buffer ‘A’ for 15 min each, followed by washing with distilled water. Ultrathin sections were post-stained with 1% KMnO_4_ for 30 min, washed in three changes of distilled water for 10 min each. All sections were examined under a transmission electron microscope (TEM, JEOL JEM 2100, Japan) at an accelerating voltage of 120 kV.

### Lignin analysis

Standard protocols were followed for Klason lignin content (Dence 1992) and determination of lignin composition by thioacidolysis–gas chromatography with flame ionization detection of TMS derivatives (Lapierre et al. 1995). Detailed description of the methodology has been provided in Method S1.

### Statistical analysis

Student’s *t* test was carried out to determine statistically significant differences of anatomical and biochemical parameters at 0.05 confidence level using Sigmastat software (Version 3.5, San Jose, CA, USA).

## Results

### Isolation and sequence characterization of *CAld5H* cDNA and genomic clones

**Fig. 1:**
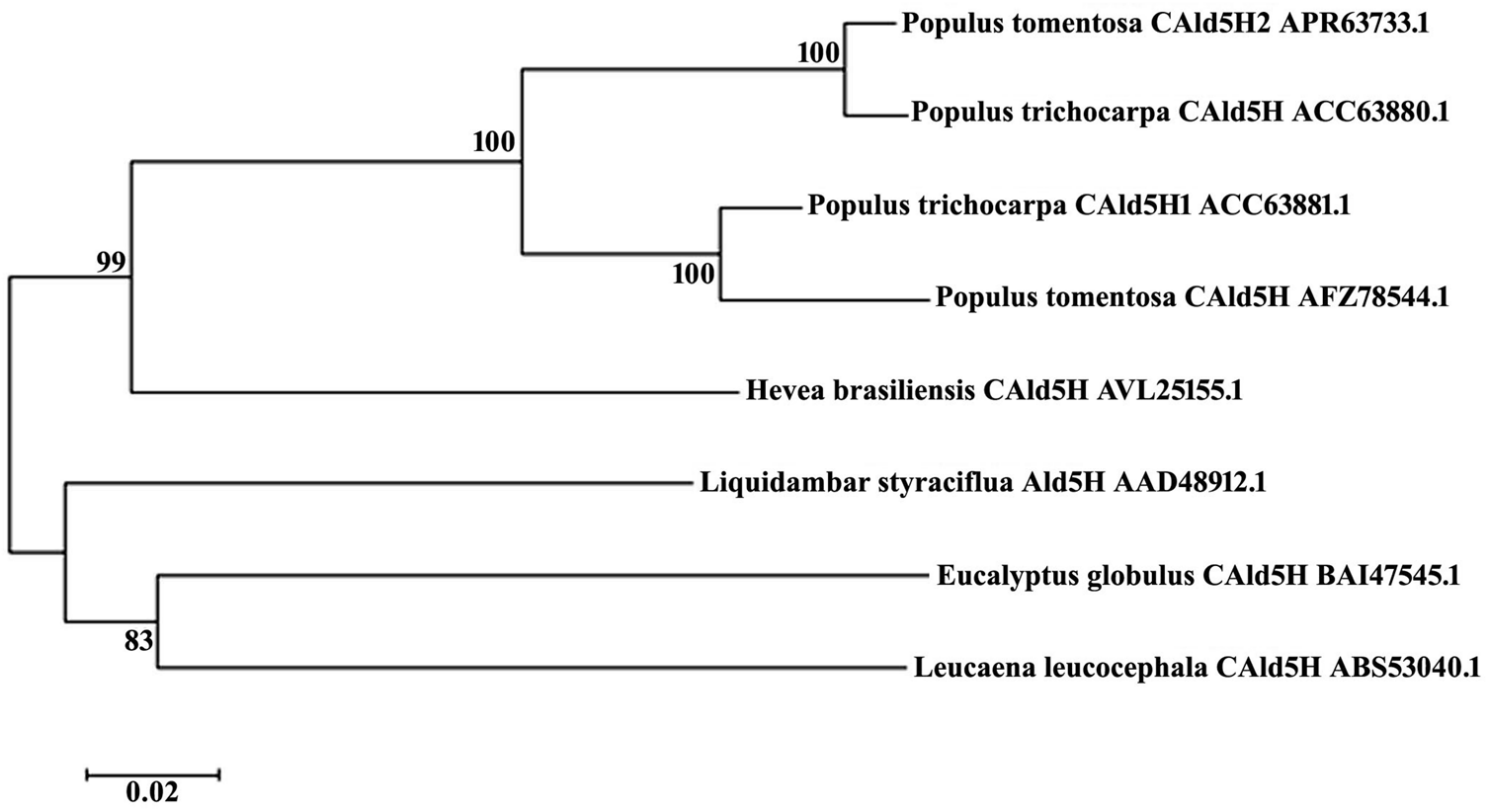
Phylogenetic analysis of predicted HbCAld5H1 protein with known sequences of CAld5H protein of other species

**Fig. 2:**
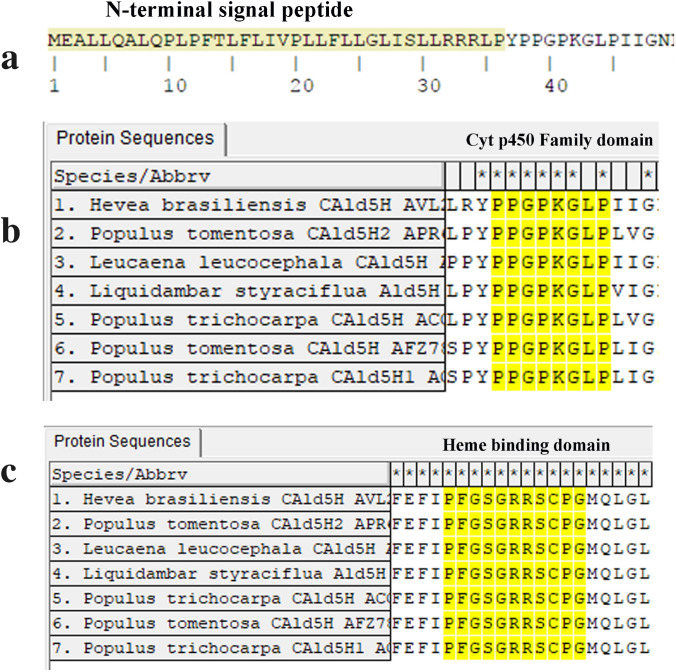
The predicted amino acid sequence of HbCAld5H1 showing N-terminal signal peptide (**a**). The multiple sequence alignment of predicted protein showing conserved domains of cytochrome p450 family, the proline-rich domain (**b**) and conserved heme-binding ligand sequence (**c**). APR63733.1: *Populus tomentosa* CAld5H2, ACC63880.1: *Populus trichocarpa* CAld5H, ACC63881.1: *Populus trichocarpa* CAld5H1, AFZ78544.1; *Populus tomentosa* CAld5H, AVL25155.1: *Hevea brasiliensis* CAld5H; AAD48912.1: *Liquidambar styraciflua* Ald5H, BAJ47545.1: *Eucalyptus globulus*, ABS53040.1: *Leucaena leucocephala*

Both the genomic DNA fragment and cDNA encoding *CAld5H* were PCR amplified, cloned and characterized from *Hevea brasiliensis*. The genomic DNA was 1.93 kb in size with one intron of 393 bp. PCR products of cDNA showed two isoforms of the gene having a size of 1.9 kb and 1.5 kb (Fig. S2). Both full length genomic and cDNA sequences were submitted to the NCBI GenBank under the accession numbers KY930624 and KY930625, respectively. The *HbAld5H* nucleotide showed 80% sequence homology with *Populus trichocarpa CAld5H* gene. The protein prediction from *Hevea* genome using FGENESH revealed that *HbCAld5H1* encodes a protein of 513 amino acids with a molecular weight of 57.05 kDa and the theoretical pI (isoelectric point) of the protein is 6.25. Encoded amino acid sequence of *HbCAld5H1* searched against the NCBI database showed the existence of a putative conserved domain, cytochrome P450, which belongs to CrypX superfamily involved in biosynthesis, transport, and catabolism of secondary metabolites. Phylogenetic analysis of the gene sequence showed homology with a number of sequences from plant species including *F5H* and other cytochrome family genes. Multiple sequence alignment of *HbCAld5H1* with reported *CAld5H* sequences revealed identities ranging from 70–80%. The amino acid sequence showed 82% sequence identity with *CAld5H1 *gene of *Populus trichocarpa* (Fig. 1). The *CAld5H* protein sequence displayed all the characteristic features of a plant P450 protein including heme-binding ligands (PFGSGRR) towards C terminus, proline-rich sequence motif PPGPKGLP, stop transfer sequence etc. (Fig. 2). Though *HbCAld5H1* has high similarity with P450 genes such as ferulate-5-hydroxylase (F5H), the N-terminal 34 amino acid sequence of *HbCAld5H1* is highly divergent from the F5H as it contains a hydrophobic region typical of the uncleavable signal peptide for anchoring P450 protein to the endoplasmic reticulum membrane (Fig. 2). The relative expression of *HbCAld5H1* was analysed in different tissues of *Hevea* using RT-PCR and a comparison was made by keeping leaf as reference (Fig S4). Transcript levels of *HbCAld5H1* was relatively more in the 2nd–3rd internodal region (IN-1) where secondary growth is just in the beginning stage and in internodes 4–5 (IN-2 with complete ring of secondary xylem).

### Molecular characterization of transformants

The PCR amplification of transgenes from the genomic DNA of all positive transformants showed bands of 1542 bp for *HbCAld5H1* sense, 842 bp for CAS-A and 484 bp for CAS-B transgenes (Fig. S2). The same were not noticed in WT plants. The marker genes, *GUS* and *hptII* (hygromycin) were also amplified from transgenic lines using gene specific primer for these genes (Fig. S2). The autoradiogram generated after exposing the Southern blot to the X-ray film showed hybridization signal for each putative sense and antisense *HbCAld5H1* transgenic plant samples (Fig. S3) except in WT plants. The hybridization signals on the blot indicated the gene integration in all transgenics.

### RT-PCR analysis and *CAld5H* gene expression in transgenic tobacco

**Fig. 3:**
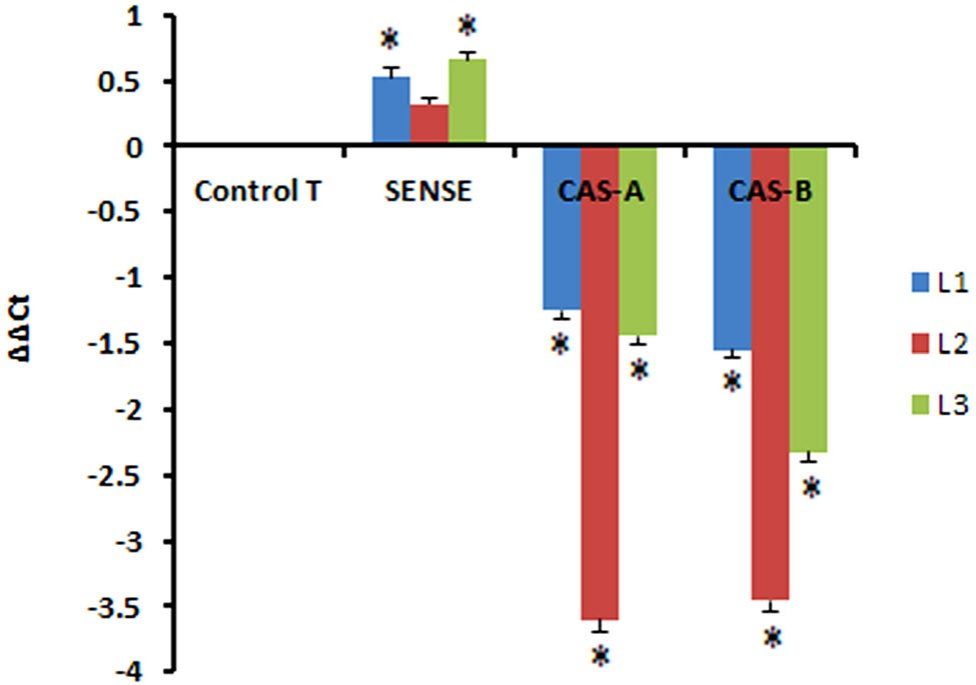
Relative expression of *CAld5H1* gene in sense and antisense (CAS-A and CAS-B) transgenic lines (L1, L2, L3) of *N. tabacum.* The statistically significant changes are represented by asterisk (*P* ≤ 0.005). *Control* untransformed wild-type tobacco

RT-PCR analysis and *CAld5H* gene expressions were performed on 3–4-month-old, three independent lines each of *CAld5H* sense antisense (CAS-A and CAS-B) along with untransformed controls acclimatized in the green house. RT-PCR displayed less transcript levels in antisense lines compared to sense lines in the leaf tissue (Fig. 3). This indicates the down- and up-regulation of *CAld5H* gene, respectively, in transgenic lines. All the three lines each of CAS-A and CAS-B transgenic lines exhibited different amounts of reduction levels in *CAld5H* expression compared to WT. The reduction in gene expression level was more apparent in CAS-B (473 bp) compared to CAS-A (842 bp) suggesting the reduced length of antisense RNA may be more effective in suppression of gene expression. A variation in degree of silencing of *CAld5H* gene expression was also evident in individual transgenic lines generated using the same antisense construct either CAS-A or CAS-B.

### Phenotypic changes in *CAld5H* sense and antisense transgenic tobacco lines

**Fig. 4:**
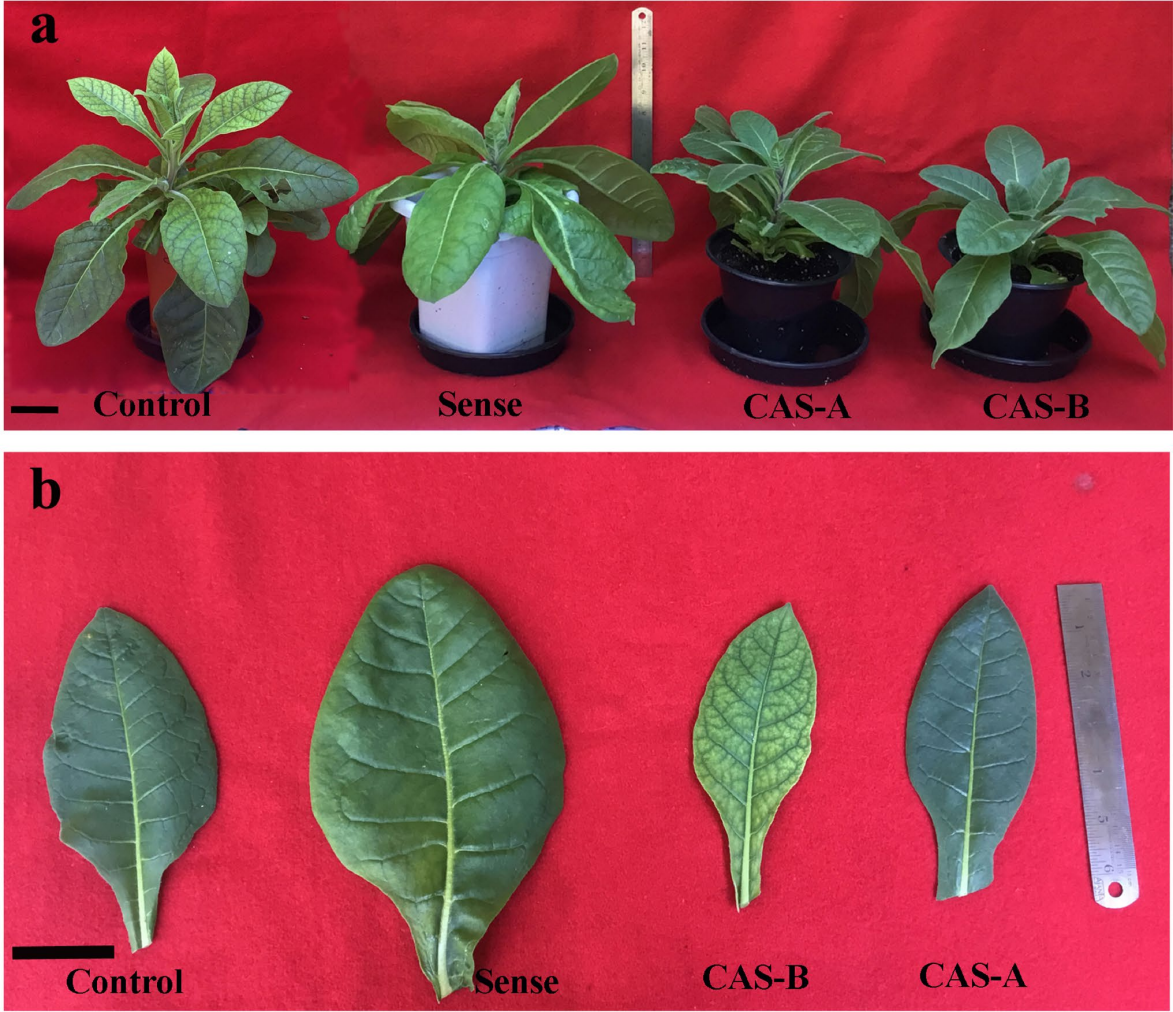
Phenotypic changes (**a**) and leaf morphology (**b**) of control, sense, antisense A (CAS-A) and antisense B (CAS-B) lines of transgenic tobacco plants. Scale bar = 2 cm

The primary transformants did not show much phenotypic variation during in vitro development. However, after transfer to green house, the transgenic plants showed distinct variations in leaf morphology. The sense plants showed an increase in leaf size, in contrast, it was reduced significantly in antisense plants compared to that of WT plants (Fig. 4). The sense plants exhibited robust leaf growth and development compared to antisense lines.

### Anatomy and lignification pattern in the leaf tissue

**Fig. 5:**
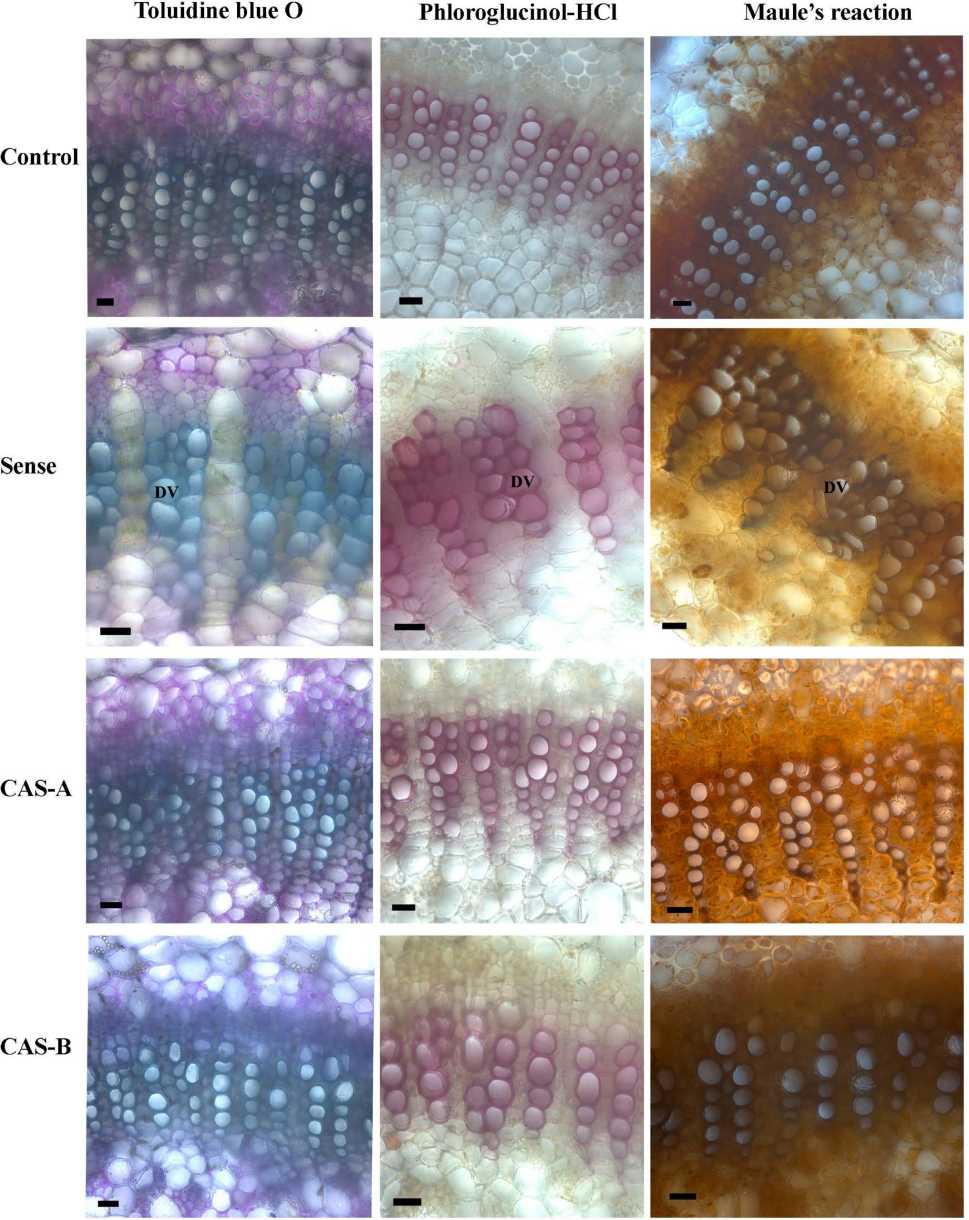
The transverse sections of leaf tissue from control and transgenic lines (Sense, CAS-A and CAS-B) stained with toluidine blue O for general histology, Weisner reaction for lignification pattern and Maule’s reaction for syringyl lignin distribution. Note the greater number of radial rows of primary xylem elements with thin secondary wall and less lignin content in sense plants compared to that of control plants while thicker secondary walls with more guaiacyl lignin units in the xylem elements of antisense lines. *DV* deformed vessel. Scale bar = 25 µm

The transverse sections from the leaf mid rib tissue of WT and transgenic lines stained with toluidine blue revealed the lignified vascular tissue consisting of protoxylem and metaxylem elements (Fig. 5). Although sense plants manifested a greater number of xylem elements in radial rows, their cell walls were thinner and often deformed in comparison with that of control and antisense lines. The cell wall of xylem vessels appeared wavy or pointed at corners compared to intact round to oval-shaped vessels found in the control plants. Weisner reaction revealed the relatively thicker, lignified secondary walls in the primary xylem elements of antisense plants (both CAS-A and CAS-B lines), however, the sense line showed thin cell walls with less lignin. Maule’s reaction of these tissues revealed the presence of guaiacyl units in the thicker secondary cell walls of proto- and metaxylem elements of WT and antisense lines (Fig. 5). The thinner secondary walls of primary xylem elements in the sense plants also displayed presence of guaiacyl lignin. These results suggest that the amount of guaiacyl lignin units is reduced by decreasing the secondary wall proportion of primary xylem elements in the vascular tissue of leaf tissue when *CAld5H* gene was overexpressed. On the contrary, the increase in number of radial extents of xylem elements in the sense plants suggest enhanced xylogenesis and vascular tissue differentiation with a greater number of cells and less secondary cell wall volume. The primary functions of leaf tissue (photosynthesis and transport of primary metabolites) are performed by chlorenchymatous and sieve cells while mechanical and conductive functions considered of secondary importance. Therefore, enhanced meristematic activity and vascular tissue differentiation could be better for growth and development of leaf tissue in relation with their specific functional dynamics compared to stem and root tissues. On the other hand, it is vital to examine the effect of *CAld5H* overexpression on secondary growth in stem tissue where mechanical and conductive functions are of priority in nature.

### Anatomy and lignification pattern in the stem

**Fig. 6:**
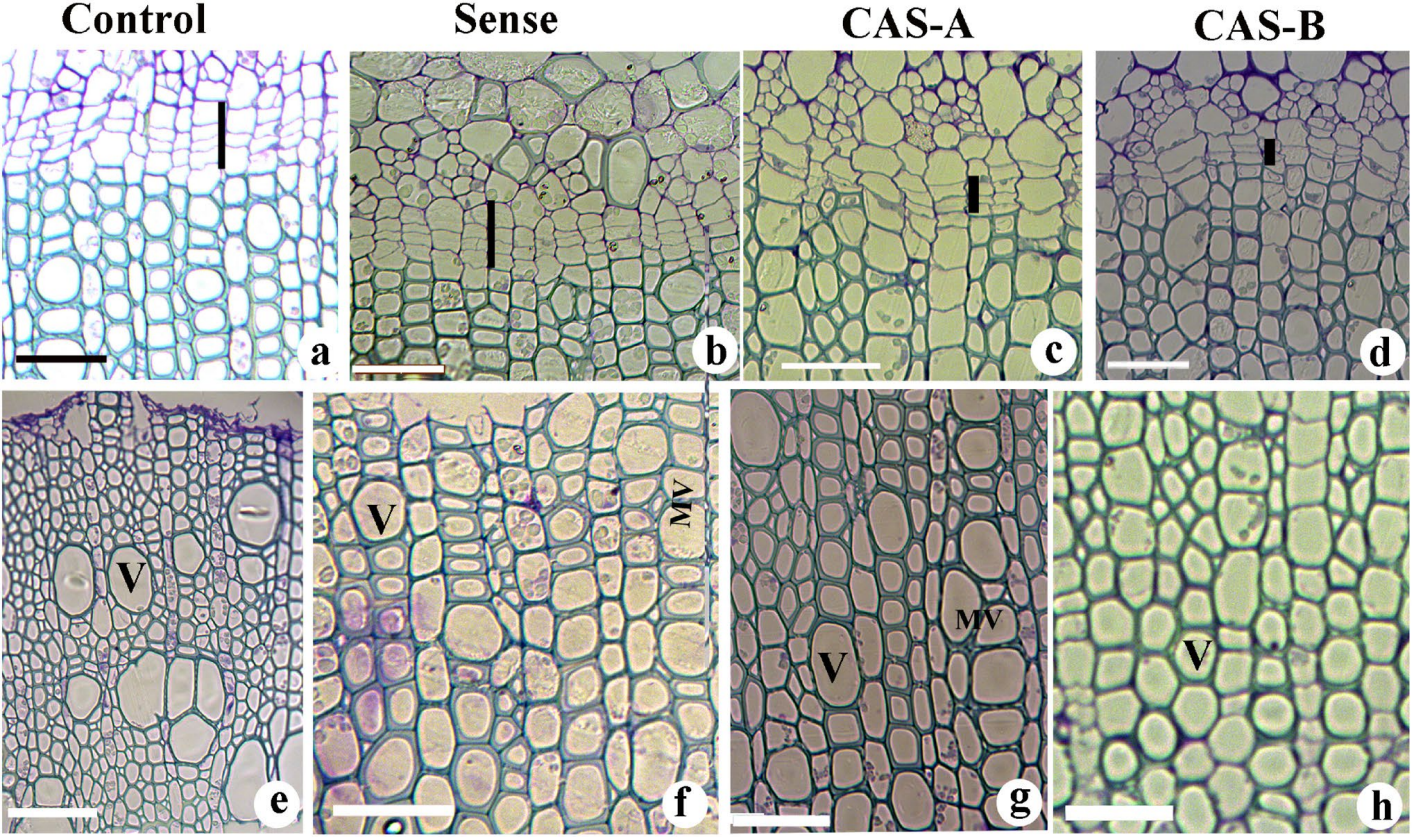
**a**–**h** Light microscopy of transverse section of the stem tissue of control and transgenic lines of *N. tabacum* showing changes in cambial zone cells (**a**–**d**) and secondary xylem tissue (**e**–**h**) characteristics. Note the wide cambial zone (vertical bar) in control (**a**) and sense plants (**b**) compared to narrow zone in CAS-A (**c**) and CAS-B (**d**) lines. The secondary xylem of control plant (**e**) showing the presence of solitary vessel compared to thick walled narrow vessels in sense plants (**f**). Note the thin walled fibers and multiple vessels in the antisense lines (CAS-A and CAS-B). *V* Vessels, *MV* multiple vessels. Scale bar = 50 µm

**Fig. 7:**
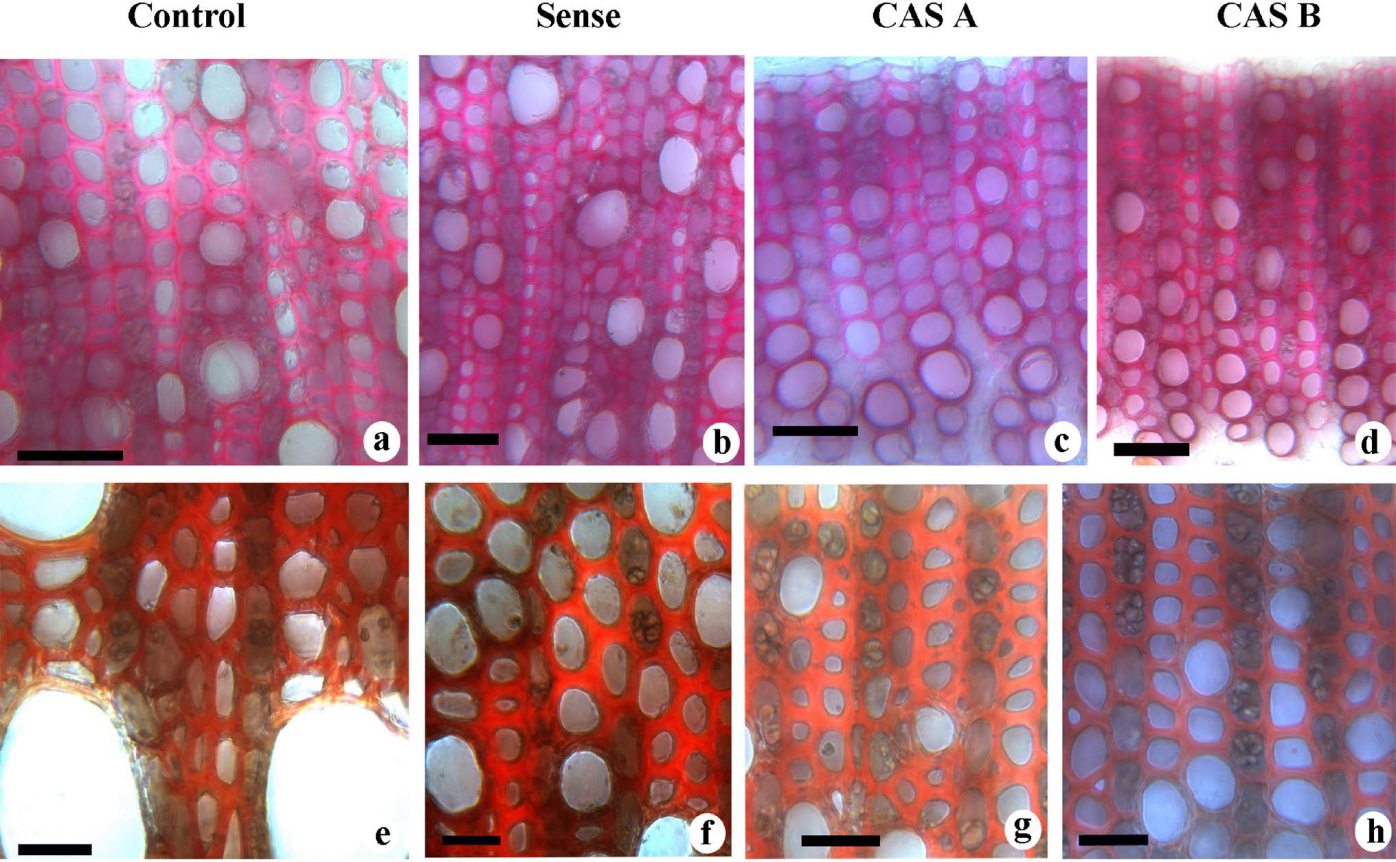
**a–h** Transverse sections from the stem stained with phloroglucinol–HCl (**a**–**d**) and Maule’s reaction (**e**–**h**) showing variation in lignin distribution pattern between control and transgenic lines of *N. tabacum.* Control plants showing high amount of lignin (**a**) with more guaiacyl monolignol units (**e**) in the vessel cell wall of control plants. Note the increase in secondary wall thickness (**b**) and more amount of syringyl lignin units (**f**) in the sense lines. The increase in vessel density (**c**, **d**) and decrease in syringyl lignin distribution in xylem elements of antisense lines CAS-A (**c**, **g**) and CAS-B (**d**, **h**). Scale bar; **a**–**d** = 50 µm; **e**–**f** = 25 µm

The cambial zone of WT plants consisted of 3–5 cell layers. The sense plants showed 3–4-layered cambial zone in which active anticlinal and periclinal divisions were evident (Fig. 6). The reduced number of cambial cell layers in CAS-A and CAS-B (1–2 cambial cell layers) suggest cambial activity and xylogenesis has been reduced in *CAld5H* antisense lines (Fig. 6). The tissue composition and cell wall thickness of xylem cells also exhibited a significant change between WT and transgenic lines. The secondary xylem showed high density of solitary vessels in WT plants while transgenic lines revealed higher density of multiple vessels (group of vessels in radial or tangential row). The sense lines showed thick walled fibres, in contrast to significantly reduced secondary wall thickness in antisense lines (Fig. 6) in comparison with WT plants. The dimensional changes in the xylem cells showed a high variation among different transgenic lines (Table 1). The fibre length and vessel element size increased significantly in sense plants while it was reduced in antisense lines especially in CAS-B lines (Table 1). Vessel width also decreased in antisense lines suggesting cell wall expansion and elongation of xylem elements has been altered due to decreased expression of *CAld5H* gene. The anatomical data suggest that the change in the extent of expression of *CAld5H* gene has impacted important stages of xylogenesis such as cambial cell division, cell expansion and secondary wall deposition. While Weisner reaction revealed that the vessel cell wall has more staining intensity (Fig. 7a), a negative reaction for Maule’s reaction suggests relative richness of guaiacyl lignin units in these cells. On the other hand, fibre cell wall showed positive colour reaction (red colour) from fibres secondary wall due to the presence of more syringyl monolignol units (Fig. 7e). An increase in lignified secondary wall region (Fig. 7b) rich in syringyl units (Fig. 7f) was noticed in the sense lines. The antisense lines displayed an increase in the vessel density with more guaiacyl lignin units (Fig. 7c, d). The fibre cell wall in the antisense lines also exhibited relatively weak response to Maule’s reaction (Fig. 7g, h) suggesting the decrease in syringyl monolignol biosynthesis following down-regulation of *CAld5H* gene.

**Table 1:** Anatomical characteristics of xylem elements in the control and transgenic tobacco lines. Measurements are taken in µm

Sample	Fibre length	Fibre width	Vessel element Length	Vessel element width
Control 1	580 ± 15	18.5 ± 1	350 ± 18	25 ± 2
Control 2	560 ± 21	19.2 ± 0.8	385 ± 12	28 ± 2.2
Sense 1	552 ± 30	19.8 ± 0.7	410 ± 36*	30 ± 4*
Sense 3	678 ± 18*	19 ± 2	485 ± 32*	31 ± 3.5*
CAS A1	439 ± 25*	17 ± 1*	305 ± 16*	21 ± 1.5*
CAS A2	535 ± 25*	19 ± 0.9	320 ± 22	32 ± 2.8*
CAS B1	502 ± 23*	18 ± 2	166 ± 10*	19.8 ± 0.7*
CAS B4	475 ± 22*	17 ± 2*	157 ± 20*	19.8 ± 0.75*

Significantly different values are represented by asterisk (*) and mean values are significantly different at *P* ≤ 0.05

### Transmission electron microscopy

**Fig. 8:**
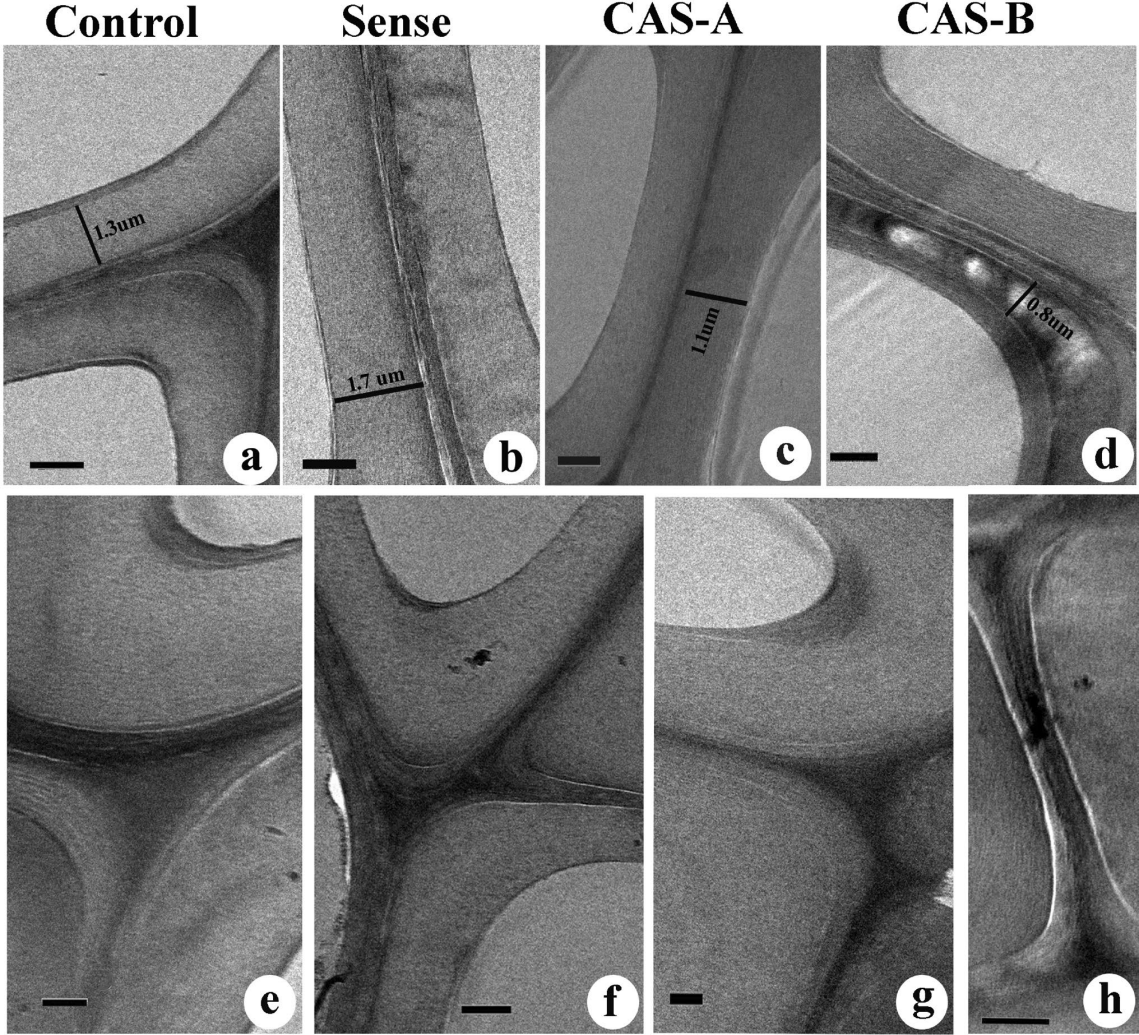
**a–h** Transmission electron micrographs from the transverse section of secondary xylem fibres in the stem tissue of control and transgenic lines of *N. tabacum* showing lignin distribution in the secondary wall (**a**–**d**) and compound middle lamella regions. Note the relatively more lignin distribution in SW and compound middle lamellae of control (**a**, **e**) and sense (**b**, **f**) plants compared to that of antisense lines. The antisense line CAS-B is showing electron translucent areas in the S2 layer of SW (**d**) representing depletion of lignin distribution in these regions. Note the compound middle lamellae of CAS-B line showing electron dense region (**h**). Scale bar = 1 µm

The ultrathin sections contrasted with KMnO4 revealed the variation in distribution between WT and transgenic lines of tobacco. The secondary xylem fibres showed maximum lignin distribution in the compound middle lamellae (CML), S1 and S3 layers (Fig. 8a, e). The thickness of S2 layer increased in the sense lines alongside lignin distribution in these wall layers compared to that of WT (Fig. 8b). The CML region also exhibited relatively higher lignin distribution in sense plants (Fig. 8f). The CAS-A lines revealed a reduction in the thickness of secondary wall (SW), especially the volume of S2 layer decreased significantly. Lignin distribution was mainly confined to the cell corners and CML region (Fig. 8c, g) and decreased lignin concentration was noticed in the secondary wall (Fig. 8c). The CAS-B antisense lines showed decrease in secondary wall thickness and presence of large electron translucent region in the S2 layer (Fig. 8d) suggesting very specific difference in spatial distribution of lignin. The CML and cell corner regions appeared electron dense in CAS-B lines (Fig. 8h) suggesting that the secondary wall lignification is affected by down-regulation of *CAld5H*.

### Immunolocalization of xylans

**Fig. 9:**
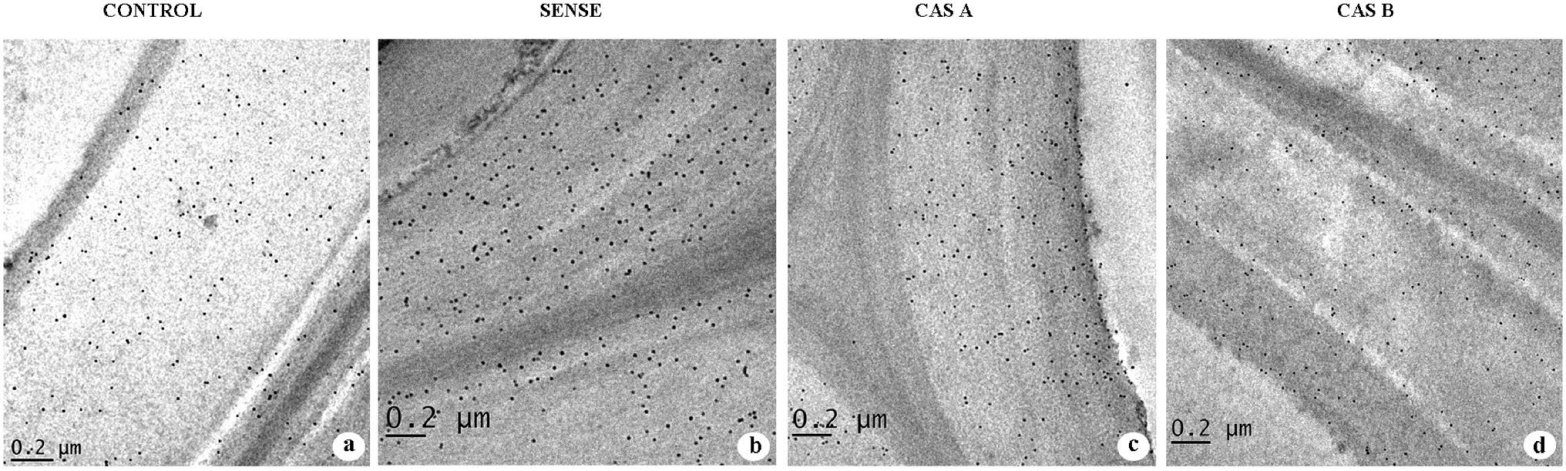
**a–d** Immunolocalization of less substituted xylans (LM10 antibody) in the cell wall of fibres from stem tissue of control and transgenic lines of *N. tabacum.* Note the decrease in gold particle density in the S2 layer of secondary wall in CAS-B line (**d**). Scale bar = 1 µm

**Fig. 10:**
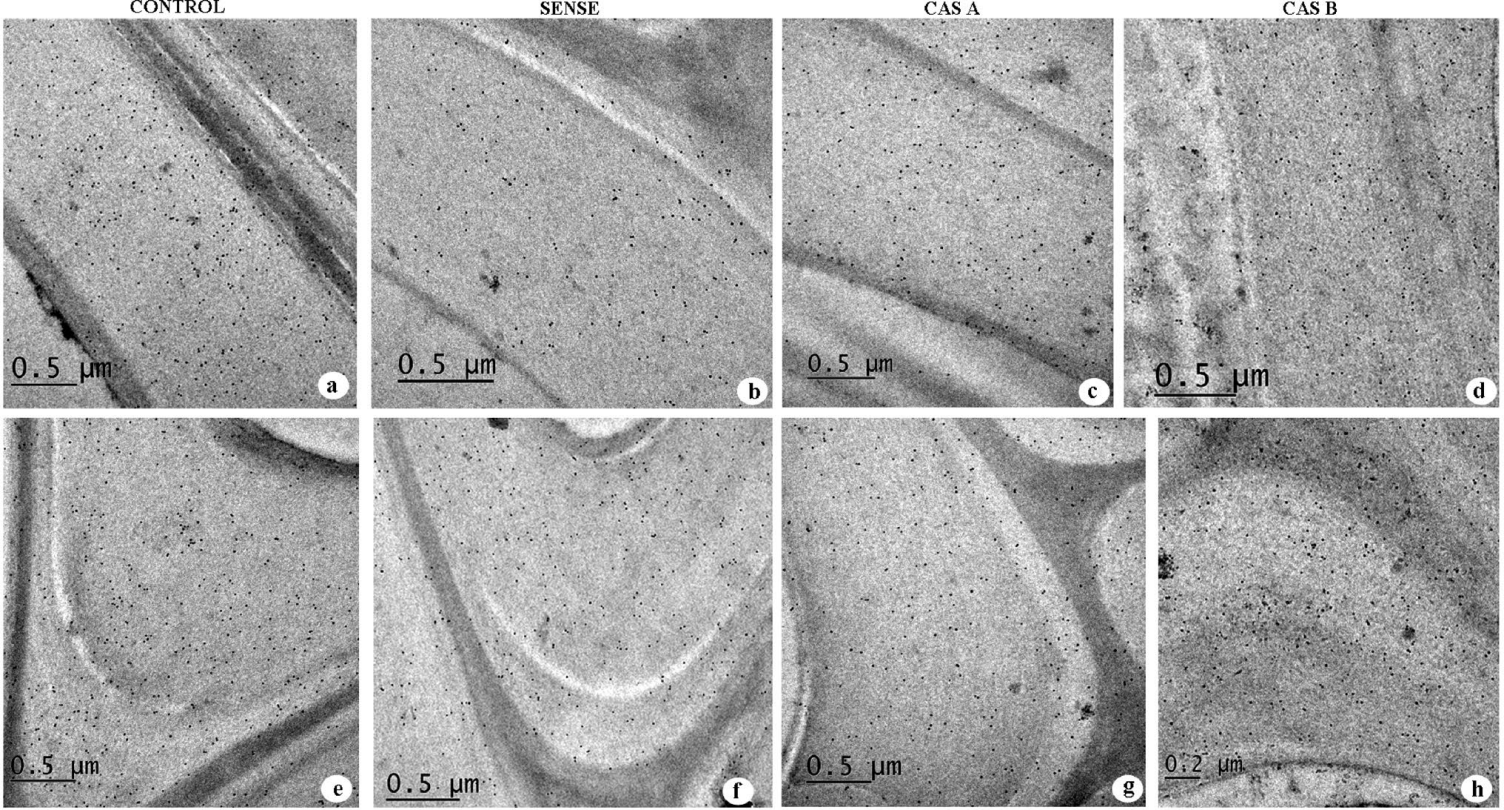
**a–h** Immunolocalization of less substituted xylans (LM11 antibody) in the cell wall of fibres from stem tissue of control and transgenic lines of *N. tabacum.* Note the significant change in density of gold particles in the bending region of secondary cell wall (**e**–**h**) between control and transgenic lines. Scale bar = 1 µm

Immunoelectron microscopy using LM 10 and LM 11 antibodies revealed the variation in distribution of less and highly substituted xylans in the secondary wall of xylem fibres in WT, sense and antisense lines of *N. tabacum*. The low substituted xylan displayed a uniform distribution throughout the secondary wall of control plants (Fig. 9a). The sense plants showed an increase in the density of gold particles suggesting an increase in xylan distribution following overexpression of *CAld5H* gene. Contrarily, antisense lines revealed a decrease in gold particle density (Fig. 9c, d). Variation in xylan distribution was more apparent in the S2 layer of fibre secondary wall of CAS-B lines in which very low density of gold particle distribution was evident. The LM11 labelling revealed that the distribution of gold particles in the secondary wall were relatively abundant in the bending region near cell corners of WT plants (Fig. 10a, e). No significant variation was noticed in the distribution pattern of gold particles between WT and transgenic lines (both sense and antisense lines) suggesting highly substituted xylans are less affected with the regulation of *CAld5H* gene (Fig. 10a–h).

### Lignin content and monomeric composition

The Klason lignin content did not show any significant variation. While the increase in total lignin content between WT and sense lines was about 5%, the reduction between WT and antisense 2–5% (Table 2). Thioacidolysis data revealed the relative yield of S lignin in sense lines compared to that of G lignin. In antisense lines, a decrease in S lignin content was noticed (Table 2). Therefore, the S/G ratio has increased in sense lines (1.74–1.9), while it decreased significantly in antisense lines CAS-A (1.2–1.3) and CAS-B (1.10–1.31) in comparison with WT plants (1.4).

**Table 2:** Percentage of Klason lignin and monomeric composition of lignin from control and transgenic tobacco plants

Sample	Klason lignin (%)	Monomeric composition		S/G ratio
		Guaiacyl (G)	Syringyl (S)	
Control 1	9.0	312.4	445	1.42
Control 2	8.5	330	460	1.39
Sense 1	9.0	701.8*	1221*	1.74*
Sense 2	9.5	421.9*	805.3*	1.91*
CAS A-1	8.0	305.8*	409.9*	1.34
CAS A-2	8.5	363.4*	436.9*	1.20*
CAS B-1	9.0	497.0*	655.1*	1.31*
CAS B-4	9.25	401.1*	445.0	1.10*

Significantly different values are represented by asterisk (*) and mean values are significantly different at *P* ≤ 0.05

## Discussion

Molecular characterization of the nucleotide and amino acid sequences of *HbCAld5H1* gene showed significant level of homology with other known sequences from dicotyledonous trees such as *P. trichocarpa*. *Hevea CAld5H* also showed a high sequence identity with three rice *CAld5Hs* isoforms (Takeda et al. 2017). This suggests that the *CAld5H* sequences are highly conserved among different families of monocots and dicots. The high expression levels of *HbCAld5H1* in different tissues of *H. brasiliensis* undergoing active cell division and differentiation such as internodes of 1-year-old stem and 20-year-old main trunk regions suggest the active role of this gene in xylogenesis. The structure and chemistry of xylem tissues in tobacco has several characteristics similar to that of wood from dicotyledonous trees. Tobacco plants produce considerable amount of secondary xylem containing vessels, fibres (both libriform and fibre tracheids), axial and ray parenchyma. Therefore, tobacco is considered as a good model system to study wood formation and cell wall chemistry in higher plants. The lignin biosynthetic pathway genes in tobacco have not been completely identified and annotated. The bioinformatics analysis of *HbCAld5H1* against whole genome shotgun assembly and transcriptome data of *N. tabacum* showed high similarity regions. The mRNA sequence of cytochrome P450.84A family gene (NM 0013256668.1) from tobacco showed the highest similarity to *HbCAld5H1* (77%) and also exhibited homology with F5H genes from *Lycopersicum esculentum* (AF150881.1) and *Camptotheca acuminata* (AY621153.1). Thus, the sequence homology analysis of whole genome and transcriptome data from tobacco strongly suggest the presence of *CAld5H* gene. The conserved region present in the *HbCAld5H1* gene used for antisense construct showed more than 90% similarity with cytochrome P450 family gene sequence of tobacco.

### Phenotypic changes associated with *HbCAld5H1* gene manipulation is not detrimental to plants

Manipulation of *CAld5H* gene in transgenic tobacco induced some morphological changes in the leaf and stem but those changes were not severe to cause any serious impact on plant growth and development. Reduction in lignin content can cause severe negative impacts on plant growth and phenotype. Down-regulation of lignin biosynthetic pathway genes often resulted in the reduction of lignin and the plants showed high vulnerability to embolism, infertility, stunted growth, shoot dieback and mortality (Voelker et al. 2011; Bonawitz and Chapple 2013). Genetic manipulation of lignin biosynthetic pathway resulted in high degree of changes in S/G monolignol composition which does not compromise plant fitness (Reddy et al. 2005; Stewart et al. 2009; Bonawitz and Chapple 2013; Anderson et al. 2015). To find a mechanistic explanation for the growth phenotypes from normal to highly dwarfed, resulting from lignin modification, Ha et al. (2019) performed a combined transcriptome and metabolome analysis in *Medicago truncatula*. Their data revealed that there were no altered transports or accumulation of toxic compounds in such plants where lignin modifications were noticed. They suggested that alteration in resource allocation is the key for such phenotypic differences. Although *HbCAld5H1* modulation did not cause any serious defects leading to mortality, certain phenotypic changes in the morphology of leaves (broad and thick leaves in sense opposed to small leaf size in down-regulated lines) and stem thickness were noticed in transgenic tobacco lines. Takeda et al. (2017) reported a visible growth depression after overexpression of *Oryza sativa Coniferaldehyde-5-hydorxylase1* gene (*OsCAld5H1)* in rice. The expression pattern of two forms of *HbCAld5H* in different tissues of *H. brasiliensis* showed relatively a higher transcript level in the leaf tissue (our unpublished data). Therefore, we postulate that the morphological changes in the leaf could be associated with changes in the transcript level after down-regulation or up-regulation of *HbCAld5H1*. Anatomical studies on stem and leaf also suggested a higher meristematic activity after overexpression of *CAld5H1* gene in tobacco. However, the exact mechanism of control over meristematic activity in leaf and stem tissues by *CAld5H1* remains obscure.

### The *CAld5H*-overexpression regulates cambial activity, xylogenesis, structure and chemistry of xylem cell wall

The important role of *CAld5H* gene expression on xylogenesis, cell wall structure and chemistry were evident from distinct changes in the anatomy and histochemistry of the leaf and stem cells of *N. tabacum.* The sense plants were characterized by the presence of a wide cambial zone with active cell divisions, more radial extent of secondary xylem, and thicker fibre secondary wall rich in syringyl lignin. On the contrary, the antisense plants displayed narrow cambial zone with less amount of xylem composed of fibres with thin secondary wall rich in guaiacyl lignin units. Previous reports on down-regulation of *CCR* and *CAD* gene expressions have also showed less cambial activity and xylem production in transgenic tobacco plants (Prashant et al. 2011; Sirisha et al. 2011). The recent investigation on wood anatomy of transgenic poplar down-regulated for lignin biosynthesis genes (CAD, C3H and C4H) revealed that the fibre wall thickness had been reduced remarkably compared to that of wild-type plants (Miller et al. 2019). Therefore, it is postulated that reduction in activity of lignin biosynthesis genes might have a direct effect on secondary wall deposition in wood tissue as it represents the major volume of lignified cell wall. It is interesting to note that the impact of gene expression on cell wall thickness is tissue specific in tobacco plants. The leaf tissue of sense plants showed an increase in xylem tissue with relatively thin cell walls compared to WT plants. The leaf xylem tissue mainly constitutes proto- and metaxylem vessel elements rich in guaiacyl lignin. The vessel cell walls rich in guaiacyl lignin is more recalcitrant than syringyl lignin and this feature believed to play an important role in their primary function of water conduction (Xu et al. 2006; Pramod et al. 2013). Syringyl lignin, on the other hand, found abundantly in the thicker secondary wall especially in fibres which is the constituent of secondary xylem. We did not detect much change in the cell wall chemistry of vessel walls in the stem tissues of both sense and antisense transgenic tobacco lines. The xylem specific expression of *Liquidambar styraciflua CAld5H (LsCAld5H)* in transgenic poplar reported to be more apparent in the fibre cells at the periphery of secondary xylem (Li et al. 2003). Therefore, *CAld5H* activity could be more specific towards syringyl monolignol composition in the fibre secondary walls. This tissue specificity also ensures the structural and functional integrity of cell wall of other cell types. The fibre specific suppression of lignin biosynthesis in F-LS transgenic poplar lines resulted in significant reduction of *CAld5H* expression compared to vessel specific suppression lines (V-LS) suggesting lignin biosynthesis genes are modified through fibre specific or vessel specific manner (Gui et al. 2020).

The TEM analysis of ultrathin sections contrasted with KMnO_4_ revealed distinct change in the lignin deposition in the secondary wall of transgenic lines. The down-regulation of *CAld5H* resulted in the reduction of S2 wall layer thickness and inhomogenous distribution of lignin within this wall layer. On the contrary, overexpression of *CAld5H* increased the secondary wall thickness and uniform lignin distribution. Up-regulation of *LsCAld5H* gene in poplar caused rapid secondary wall thickening and acceleration of syringyl lignin deposition (Li et al. 2003). The fibre specific suppression of *LTF1* gene revealed 35–45% reduction in secondary cell wall thickness of xylem fibres in transgenic poplar lines (Gui et al. 2020). Down-regulation of *LlCCR* in tobacco caused an increase in S2 layer proportion with less reactivity to KMnO4 and high reactivity to PATAg staining due to less lignin and more polysaccharide units, respectively (Prashant et al. 2011). In general, S lignin distribution is mainly confined to secondary wall of hard wood fibres and S2 layer represents the thickest layer among three SW layers. Reduction in S2 layer could be a structural consequence of syringyl lignin. As pointed out by Gui et al. (2020), lignin modification in cell specific manner can be more promising for improvement of wood biomass production without growth penalty.

### The lignin-xylan relationship

The major part of plant biomass in hard wood trees is represented by secondary cell walls of xylem fibres. In general, hemicelluloses interact with cellulose microfibrils, form a stable network while lignin interacts with hemicelluloses. However, the monomeric level organization of hemicellulose and lignin into a dynamic three-dimensional matrix structure in the secondary wall of xylem elements is poorly understood. In the present study, a significant decrease in low substituted xylan epitope labelling (ls ACG Xs) was noticed in fibre secondary wall with reduced syringyl lignin content in *CAld5H*-deficient transgenic tobacco lines. In *VND 7* mutant lines of *Arabidopsis*, the secondary cell wall assembly during ectopic protoxylem vessel differentiation is reported to be characterized by cellulose independent deposition of xylan-lignin organization (Takenaka et al. 2018). The study on physical nature of lignin interactions with other wall polysaccharides using solid state NMR spectroscopy also revealed the existence of abundant electrostatic interaction between lignin with the polar motifs of xylan (Kang et al. 2019). During enzymatic hydrolysis, the accessibility of cellulose in secondary walls has been proposed to be significantly influenced by their xylan and lignin contents (Chang and Holtzapple 2000; Biswal et al. 2014). Davison et al. (2006) reported that the xylan acid hydrolysis yields were inversely proportional to the syringyl/guaiacyl (S/G) ratio. Our histochemical and immunolabelling results suggest the possibility of strong interaction between low substituted xylan and syringyl lignin in the secondary wall of xylem fibres. A detailed investigation on interaction of ls ACG Xs and syringyl lignin will be very useful for further genetic engineering research on cell wall chemistry aimed to optimize the post-harvest processing of lignocellulosic biomass for various industrial and commercial applications such as biofuels and biomaterials.

### *HbCAld5H1*-induced change in lignin content and monolignol composition

The down-regulation or overexpression of *HbCAld5H1* gene in tobacco did not result in any significant change in the total lignin content. On the other hand, the thioacidolysis data revealed that the syringyl lignin content has been altered. It has increased significantly in sense, while it decreased in antisense lines. Genetic manipulation of *OsCAld5H1* gene in rice plants did not alter the total lignin, while thioacidolysis and NMR analysis revealed G lignin enrichment in down-regulated lines while up-regulation resulted in S lignin enrichment in lignin (Takeda et al. 2017). In transgenic poplar, overexpression of *LsCAld5H* resulted in a 2.5-fold increase in S/G ratio without any significant influence on total lignin (Li et al. 2003). Our results are in agreement with the previous reports on the role of *CAld5H* gene in increasing S/G ratio without altering total lignin content. The increased S/G ratio is highly promising from industrial perspective, especially in improving downstream processing of lignocellullosic biomass, such as the efficiency of chemical pulping and pulp bleachability of poplar (Hutley et al. 2003) and also improved sugar fermentation both in *Arabidopsis* and poplar (Li et al. 2010; Shi et al. 2016).

The functional validation in heterologous system suggests that *HbCAld5H1* could be an ideal target gene for altering chemistry of lignocellulosic biomass in *Hevea* trees for commercial and industrial applications such as timber, biofuel, paper and pulp.

## Electronic supplementary material

Below is the link to the electronic supplementary material.

Fig. S1: The HbCAld5H1 antisense (A) and sense constructs (B). CaMV 35S Cauliflower mosaic virus 35S RNA promoter, Antisense CAld5H cDNA of *H. brasiliensis* in antisense orientation; sense HbCAld5H1 cDNA of *H. brasiliensis* in sense orientation; Nos PolyA termination sequence of the nopaline synthase gene, T Poly A termination sequence of phosphotransferase gene, GUS β-glucuronidase gene (JPG 353 kb)

Fig. S2: Agarose gel electrophoresis of PCR amplified products (A) *HbCAld5H* genomic DNA fragment (B) full length gene of *HbCAld5H1* (1.5 kb) and *HbCAld5H2* (1.9 kb) amplified from the cDNA of *H. brasiliensis* (C) control (c) and transgenic lines of sense (S 1, 2, 3), (D) *CAld5H* antisense A (CAS-A 1, 2, 3) and (E) *CAld5H* antisense B (CAS-B 1, 2, 3) (F) hygromycin (hptII) and GUS β-glucuronidase gene from genomic DNA of control (C) and transgenic lines of sense (S), CAS-A (A) and CAS-B (B) (JPG 259 kb)

Fig. S3: Southern blot analysis of control (C), *CAld5H* Sense (S1, S2, S3), *CAld5H* antisense A (CAS A, A1, A2, A3) and *CAld5H* antisense B (CAS-B, B1, B4, B5) lines of tobacco showing gene integration using *CAld5H* gene specific probe (sense probe with 1542bp and antisense probe with 842 bp for CAS-A and 473bp for CAS-B). The DNA of sense, antisense (CAS-A and CAS-B) and controls were digested with Sac I. Positive hybridization observed at the multiple sites of gene integration in putative sense while single copy of endogenous gene showed hybridization in control plants. In antisense lines hybridization was observed at multiple integration regions except in controls (JPG 401 kb)

Fig. S4: Relative expression of *HbCAldH1* between different tissues of *H. brasiliensis*. IN 1 = 2-3rd (JPG 680 kb)

Supplementary file5 (DOCX 12 kb)

Supplementary file6 (DOCX 23 kb)

## Data Availability

Datasets generated during and/or analysed in the current study are available from the corresponding author on request.

## References

[CR1] Anderson NA, Tobimatsu Y, Ciesielski PN, Ximenes E, Ralph J, Donohoe BS, Ladisch M, Chapple C (2015). Manipulation of guaiacyl and syringyl monomer biosynthesis in an Arabidopsis cinnamyl alcohol dehydrogenase mutant results in atypical lignin biosynthesis and modified cell wall structure. Plant Cell.

[CR2] Berlyn GP, Miksche JP (1976). Botanical Microtechnique and Cytochemistry.

[CR3] Biswal AK, Soeno K, Gandla ML, Immerzee P, Pattathil S, Lucenius J, Serimaa R, Hahn MG, Moritz T, Jonsson LJ, Nordstom MI, Mellerowicz EJ (2014). Aspen pectate lyase PtxtPL-27 mobilizes matrix polysaccharides from woody tissues and improves saccharification yield. Biotechnol Biofuels.

[CR4] Boerjan W, Ralph J, Baucher M (2003). Lignin biosynthesis. Ann Rev Plant Biol.

[CR5] Bonawitz ND, Chapple C (2013). Can genetic engineering of lignin deposition be accomplished without an unacceptable yield penalty?. Curr Opin Biotechnol.

[CR6] Boudet AM, Kajita S, Grima-Pettenati J, Goffner D (2003). Lignins and lignocellulosics: a better control of synthesis for new and improved uses. Trends Plant Sci.

[CR7] Bustin SA (2002). Quantification of mRNA using real-time reverse transcription PCR (RT-PCR): trends and problems. J Mol Endocrinol.

[CR8] Carocha V, Soler M, Hefer C, Wang HC, Myburg AA, Paiva JAP, Pettenati JG (2015). Genome wide analysis of the lignin toolbox of *Eucalyptus grandis*. New Phytol.

[CR9] Chang VS, Holtzapple MT (2000). Fundamental factors affecting biomass enzymatic reactivity. Appl Biochem Biotechnol.

[CR10] Chang S, Puryear J, Cairney J (1993). Simple and efficient method for isolating RNA from pine trees. Plant Mol Biol Rep.

[CR11] Chen X, Wang H, Li X, Zhan Y, Zeng F (2019). Molecular cloning and functional analysis of 4-coumarate: CoA ligase 4 (4CL-like 1) from *Fraxinus mandshurica* and its role in abiotic stress tolerance and cell wall biosynthesis. BMC Plant Biol.

[CR12] Chiang VL (2006). Monolignol biosynthesis and genetic engineering of lignin in trees, a review. Environ Chem Lett.

[CR13] Davison BH, Drescher SR, Tuskan GA, Davis MF, Nghiem NP (2006). Variation of S/G ratio and lignin content in a *Populus* family influences the release of xylose by dilute acid hydrolysis. Appl Biochem Biotechnol.

[CR14] Dence CW, Lin SY, Dence CW (1992). The determination of Lignin. Methods in lignin chemistry.

[CR15] Donaldson L (1992). Lignin distribution during late wood formation in *Pinus radiata* D.DON. IAWA Bull.

[CR16] Garcia JR, Anderson N, Feuvre RL, Iturra C, Elissetche J, Chapple C, Valenzuela S (2014). Rescue of syringyl lignin and sinapate ester biosynthesis in *Arabidopsis thaliana* by a coniferaldehyde 5 hydroxylase from *Eucalyptus globulus*. Plant Cell Rep.

[CR17] Gui J, Lam PY, Tobimatsu Y, Sun J, Huang C, Cao S, Zhong Y, Umezawa T, Li L (2020). Fibre specific regulation of lignin biosynthesis improves biomass quality in poplar. New Phytol.

[CR18] Ha CM, Fine D, Rao X, Martin MZ, Engle NL, Wherritt DJ, Tschaplinski TJ, Sumner LW, Dixon RA (2019). Ectopic gene expression is associated with growth defects in *Medicago truncatula* lignin pathway mutants. Plant Physiol.

[CR19] Hallac BB, Sannigrahi P, Pu Y, Ray M, Murphy RJ, Ragauskas AJ (2010). Effect of ethanol organosolv pretreatment on enzymatic hydrolysis of *Buddleja davidii* stem biomass. Biomass Ind Eng Chem Res.

[CR20] Huntley SK, Ellis D, Gilbert M, Chapple C, Mansfield SD (2003). Significant increases in pulping efficiency in C4H–F5H- transformed poplars: improved chemical savings and reduced environmental toxins. J Agric Food Chem.

[CR21] Kang X, Kirui A, Widange MCD, Vigier FM, Cosgrow DJ, Wang T (2019). Lignin polysaccharide interactions in plant secondary cell walls revealed by solid state NMR. Nature Comm.

[CR22] Lapierre C, Pollet B, Ronaldo C (1995). New insights into the molecular architecture of hard wood lignins by chemical degradative methods. Res Chem Intermed.

[CR23] Li X, Chapple C (2010). Understanding lignification: challenges beyond monolignol biosynthesis. Plant Physiol.

[CR24] Li L, Zhou Y, Cheng X, Sun J, Marita JM, Ralph J, Chiang VL (2003). Combinatorial modification of multiple lignin traits in trees through multigene co-transformation. Proc Nat Acad Sci (USA).

[CR25] Li X, Ximenes E, Kim Y, Slininger M, Meilan R, Ladisch M, Chapple C (2010). Lignin monomer composition affects Arabidopsis cell wall degradability after liquid hot water pretreatment. Biotechnol Biofuels.

[CR26] Meshitsuka G, Nakano J (1979). Studies on the mechanism of lignin color reaction (XIII): Maüle color reaction (9). Mokuzai Gakkaishi.

[CR27] Miller ZD, Peralta PN, Mitchell P, Chiang VL, Kelley SS, Edmunds CW, Peszlen IM (2019). Anatomy and chemistry of *Populus trichocarpa* with genetically modified lignin content. BioResources.

[CR28] Osakabe K, Tsao CC, Li L, Popko JL, Umezawa T, Carraway DT, Smeltzer RH, Joshi CP, Chiang VL (1999). Coniferyl aldehyde 5-hydroxylation and methylation direct syringyl lignin biosynthesis in angiosperms. Proc Natl Acad Sci (USA).

[CR29] Pramod S, Rao KS, Sundberg A (2013). Structural, histochemical and chemical characterization of normal, tension and opposite wood of subabul (*Leucaena leucocephala*). Wood Sci Technol.

[CR30] Pramod S, Rajput KS, Rao KS (2019). Immnolocalization of β (1–4) galactan, xyloglucan ad xylan in the reaction xylem of *Leucaena leucocephala* (Lam) de Wit. Plant Physiol Biochem.

[CR31] Prashant S, Sunitha MS, Pramod S, Rao KS, Rawal SK, Kavi Kishor PB (2011). Down-regulation of *Leucaena leucocephala* cinnamoyl CoA reductase (*LlCCR*) gene induces significant changes in phenotype, soluble phenolic pools and lignin in transgenic tobacco. Plant Cell Rep.

[CR32] Ralph J, Lapierre C, Boerjan W (2019). Lignin structure and its engineering. Curr Opin Biotechnol.

[CR33] Reddy MS, Chen F, Shadle G, Jackson L, Aljoe H, Dixon RA (2005). Targeted down-regulation of cytochrome P450 enzymes for forage quality improvement in alfalfa (*Medicago sativa* L.). Proc Natl Acad Sci (USA).

[CR34] Saitou N, Nei M (1987). The Neighbor-Joining Method-a new method for reconstructing phylogenetic trees. Mol Biol Evol.

[CR35] Sambrook J, Russell D (2001). Molecular cloning: a laboratory manual.

[CR36] Schmidt GW, Delaney SK (2010). Stable internal reference gene for normalization of real-time RT-PCR in tobacco (*Nicotiana tabacum*) durinh development and abiotic stress. Mol Genet Genom.

[CR37] Shi J, Pattathil S, Ramakrishnan P, Anderson NA, Kim JI, Venketachalam S, Hahn MG, Chapple C, Simmons BA, Singh S (2016). Impact of engineered lignin composition on biomass recalcitrance and ionic liquid pretreatment efficiency. Green Chem.

[CR38] Sirisha VL, Kumar RD, Prashant S, Pramod S, Jalaja N, Rao MP, Rao NS, Mishra P, Rao KS, Khan BM, Kavi Kishor PB (2011). Cloning, characterization and impact of up- and down-regulating subabul cinnamyl alcohol dehydrogenase (CAD) gene on plant growth and lignin profiles in transgenic tobacco. Plant Growth Reg.

[CR39] Speer EO (1987). A method of retaining phloroglucinol proof lignin. Stain Technol.

[CR40] Stewart JJ, Akiyama T, Chapple C, Ralph J, Mansfield SD (2009). The effects on lignin structure of overexpression of ferulate 5-hydroxylase in hybrid poplar. Plant Physiol.

[CR41] Takeda Y, Koshiba T, Tobimatsu Y, Suzuki S, Murakami S, Yamamura M, Rahman MM, Takano T, Hattori T, Sakamoto M, Umezawa T (2017). Regulation of coniferaldehyde 5 hydroxylase expression to modulate cell wall lignin structure in rice. Planta.

[CR42] Takenaka Y, Watanabe Y, Schuetz M, Unda F, Hill JL, Phookaew P, Yoneda A, Mansfield SD, Samuels L, Ohtani M, Demura T (2018). Patterned deposition of xylan and lignin is independent from that of the secondary wall cellulose of *Arabidopsis* xylem vessels. Plant Cell.

[CR43] Tamura K, Peterson D, Peterson N, Stecher G, Neil M, Kumar S (2011). MEGA5: molecular evolutionary genetics analysis using maximum likelihood, evolutionary distance, and maximum parsimony methods. Mol Biol Evol.

[CR44] Vanholme R, Morrel K, John R, Boerjan W (2008). Lignin engineering. Plant Biol.

[CR45] Voelker SL, Lachenbruch B, Meinzer FC, Kitin P, Strauss SH (2011). Transgenic poplars with reduced lignin showed impaired xylem conductivity, growth efficiency and survival. Plant Cell Environ.

[CR46] Xu F, Sun RC, Lu Q, Jones GL (2006). Comparative study of anatomy and lignin distribution in normal and tension wood of *Salix ordejecii*. Wood Sci Technol.

