# Supplementary figures and images for: Hevea brasiliensis coniferaldehyde-5-hydroxylase (HbCAld5H) regulates xylogenesis, structure and lignin chemistry of xylem cell wall in Nicotiana tabacum
Source: Plant Cell Rep. 2020 Oct 17;40(1):127–42. doi: 10.1007/s00299-020-02619-8 (PMC7811508; doi:10.1007/s00299-020-02619-8)

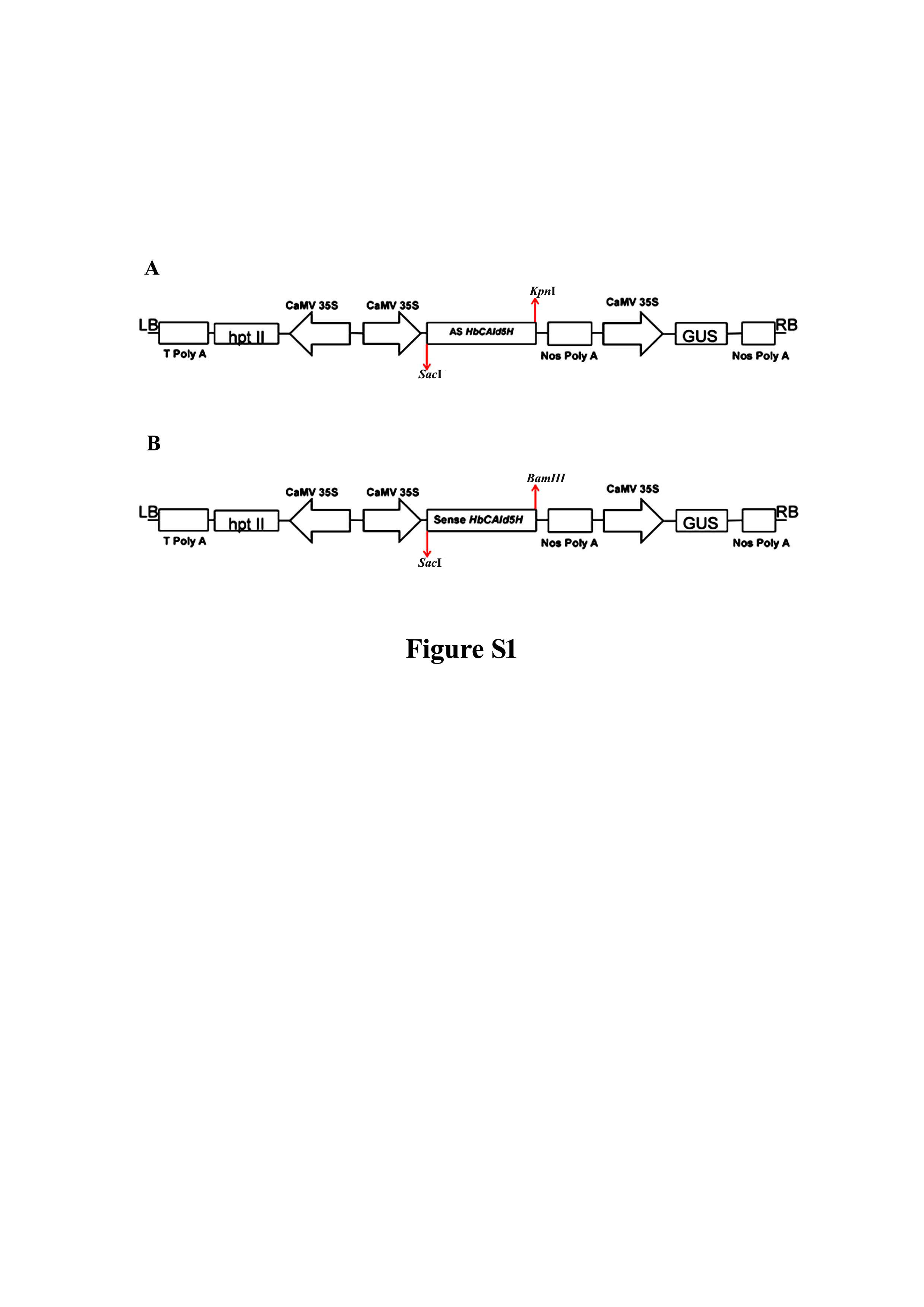

Supplement: Supplementary file 1 — Fig. S1: The HbCAld5H1 antisense (A) and sense constructs (B). CaMV 35S Cauliflower mosaic virus 35S RNA promoter, Antisense CAld5H cDNA of H. brasiliensis in antisense orientation; sense HbCAld5H1 cDNA of H. brasiliensis in sense orientation; Nos PolyA termination sequence of the nopaline synthase gene, T Poly A termination sequence of phosphotransferase gene, GUS β-glucuronidase gene (JPG 353 kb) [file 299_2020_2619_MOESM1_ESM.jpg]

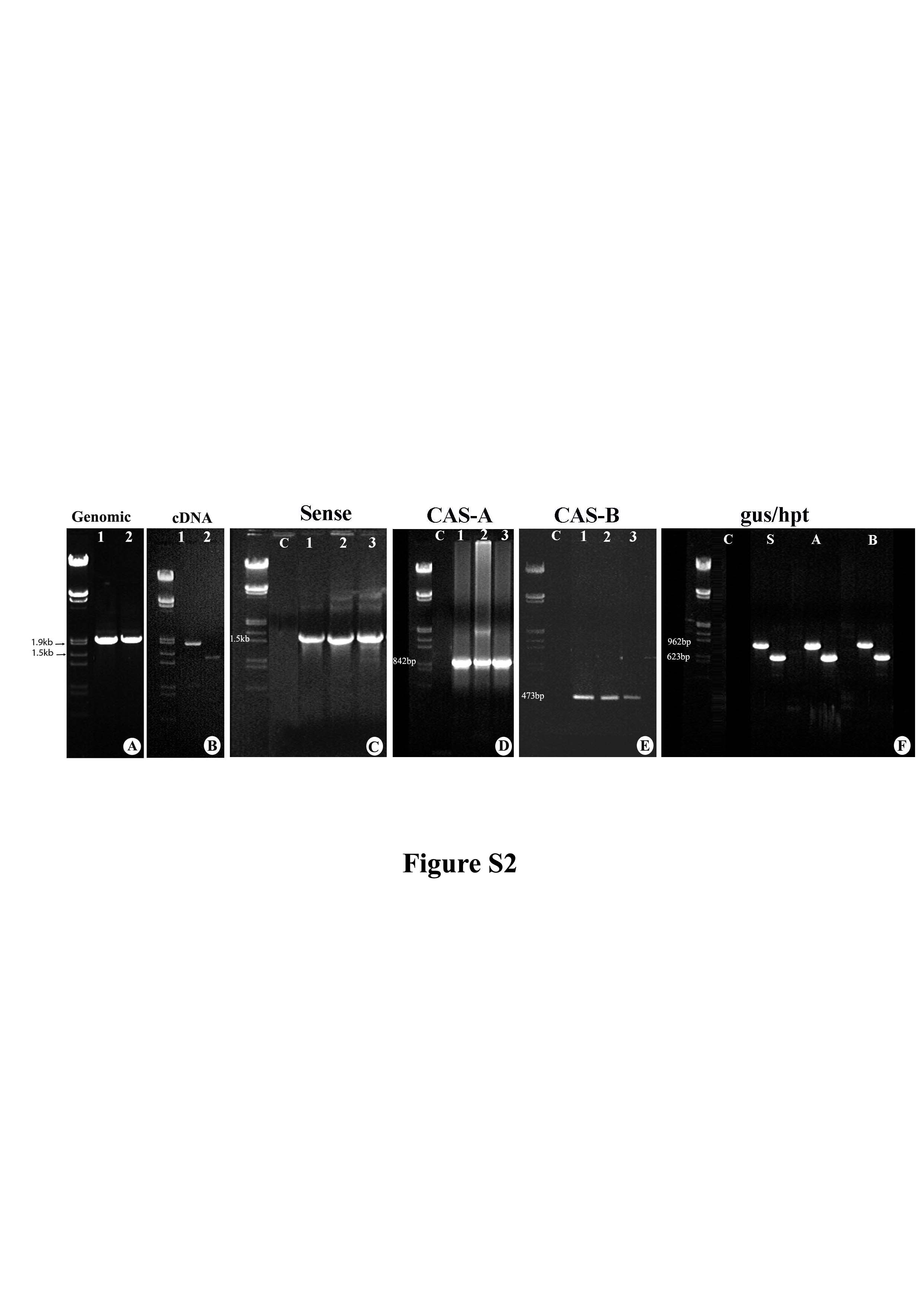

Supplement: Supplementary file 2 — Fig. S2: Agarose gel electrophoresis of PCR amplified products (A) HbCAld5H genomic DNA fragment (B) full length gene of HbCAld5H1 (1.5 kb) and HbCAld5H2 (1.9 kb) amplified from the cDNA of H. brasiliensis (C) control (c) and transgenic lines of sense (S 1, 2, 3), (D) CAld5H antisense A (CAS-A 1, 2, 3) and (E) CAld5H antisense B (CAS-B 1, 2, 3) (F) hygromycin (hptII) and GUS β-glucuronidase gene from genomic DNA of control (C) and transgenic lines of sense (S), CAS-A (A) and CAS-B (B) (JPG 259 kb) [file 299_2020_2619_MOESM2_ESM.jpg]

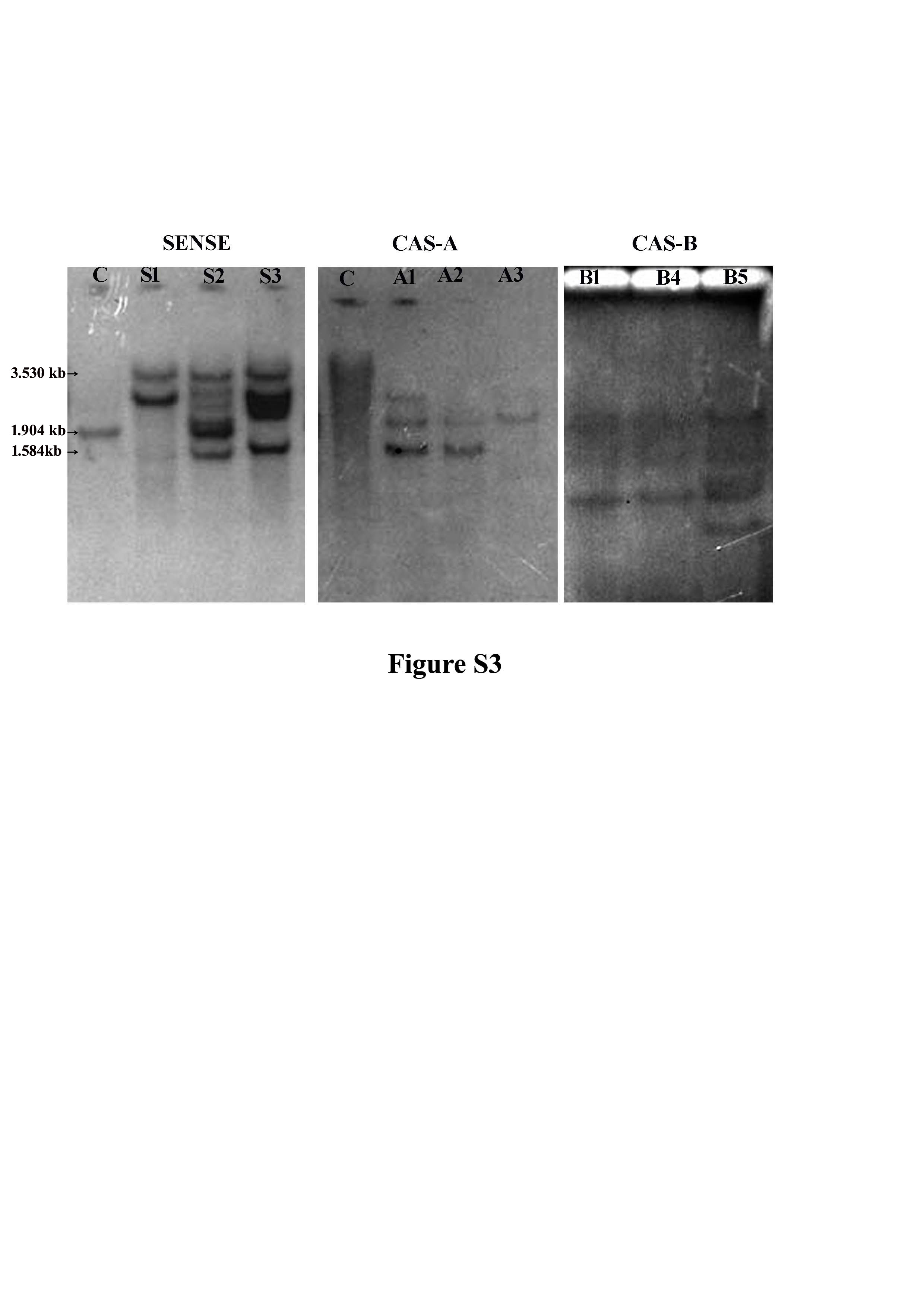

Supplement: Supplementary file 3 — Fig. S3: Southern blot analysis of control (C), CAld5H Sense (S1, S2, S3), CAld5H antisense A (CAS A, A1, A2, A3) and CAld5H antisense B (CAS-B, B1, B4, B5) lines of tobacco showing gene integration using CAld5H gene specific probe (sense probe with 1542bp and antisense probe with 842 bp for CAS-A and 473bp for CAS-B). The DNA of sense, antisense (CAS-A and CAS-B) and controls were digested with Sac I. Positive hybridization observed at the multiple sites of gene integration in putative sense while single copy of endogenous gene showed hybridization in control plants. In antisense lines hybridization was observed at multiple integration regions except in controls (JPG 401 kb) [file 299_2020_2619_MOESM3_ESM.jpg]

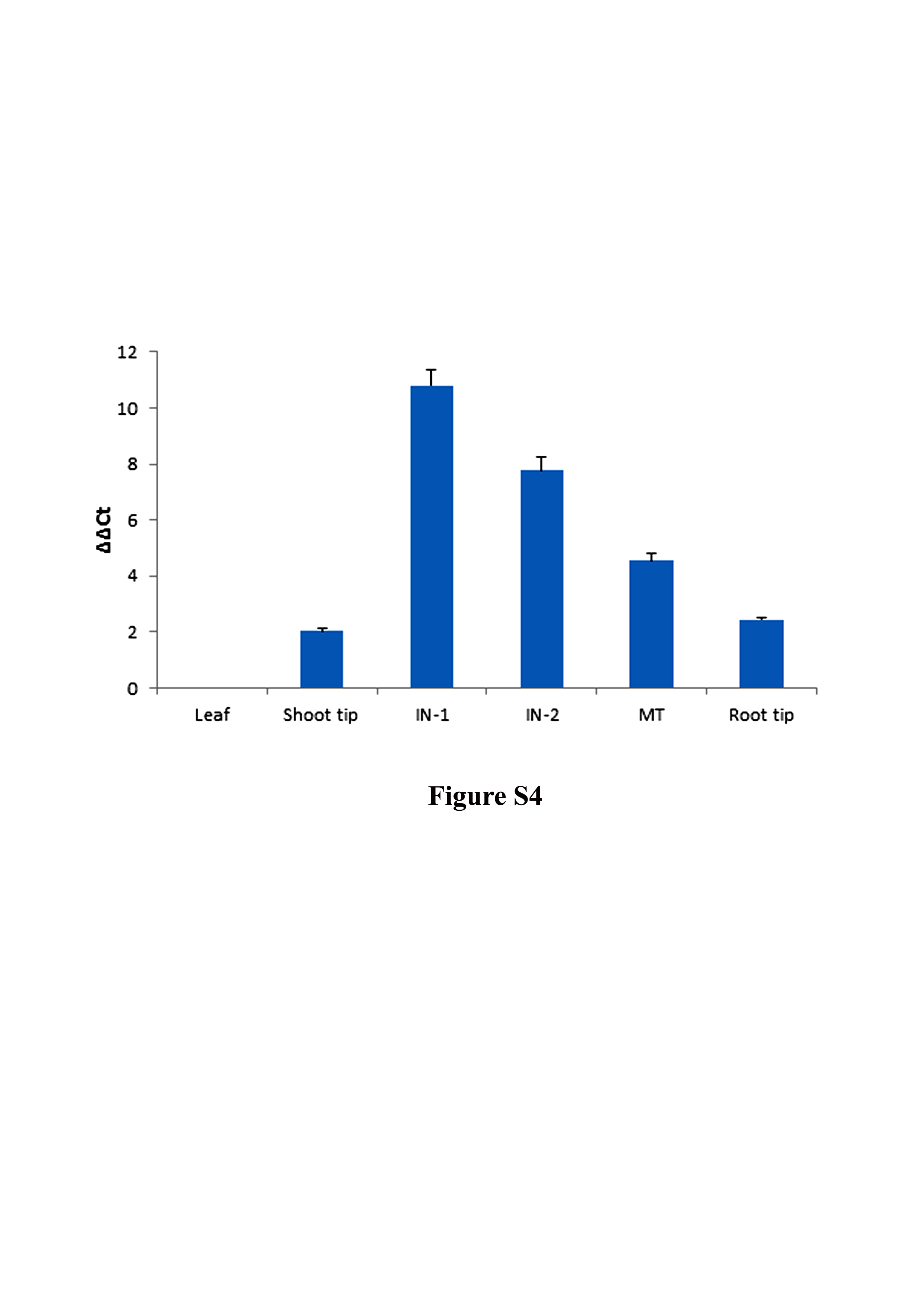

Supplement: Supplementary file 4 — Fig. S4: Relative expression of HbCAldH1 between different tissues of H. brasiliensis. IN 1 = 2-3rd (JPG 680 kb) [file 299_2020_2619_MOESM4_ESM.jpg]
