# Supplementary material for: Hevea brasiliensis coniferaldehyde-5-hydroxylase (HbCAld5H) regulates xylogenesis, structure and lignin chemistry of xylem cell wall in Nicotiana tabacum
Source: Plant Cell Rep. 2020 Oct 17;40(1):127–42. doi: 10.1007/s00299-020-02619-8 (PMC7811508; doi:10.1007/s00299-020-02619-8)
Supplement: Supplementary file 5 — Supplementary file5 (DOCX 12 kb) [file 299_2020_2619_MOESM5_ESM.docx]

Supplementary Table 1: List of primers used in this study

Genomic DNA

Forward: 5′ CCATTACATCTTGCCCAACC3’

Reverse: 5′CCTGATTCGGTTTCATCGTC3′

cDNA

Forward: 5′ GCCATGGAAGCTCTTCTCCAAG 3

Reverse: 5′CCTTTAGAGCGGGCACAACAAA3′

qPCR

Forward: -CAACAAAGGGAAGCCAGTGAA-5’

Reverse : 3’-CCATCCAAGCCAAGGAAT-5’

Sense

Forward: 5’CGGGAGCTCATGGAAGCTCTTCTCCAAGC3’

Reverse: 5’CGCGGATCCTTAGAGCGGGCACAACAAACG3’

CAld5H antisense construct A (CAS A)

Forward: 5’CGGGAGCTCTTAGAGGGGCACAACAAAC3’

Reverse: 5’CGGGGTACCCTCAACTCAAGACTTGCCAAG3’

CAld5H antisense construct B (CAS B)

Forward: 5’CGGGAGCTCGCTTGGAGAACTCCTGCAAA3’

Reverse: 5’CGGGGTACCGATGGACCAGTTAACTCACCG3’
