# Supplementary material for: Hevea brasiliensis coniferaldehyde-5-hydroxylase (HbCAld5H) regulates xylogenesis, structure and lignin chemistry of xylem cell wall in Nicotiana tabacum
Source: Plant Cell Rep. 2020 Oct 17;40(1):127–42. doi: 10.1007/s00299-020-02619-8 (PMC7811508; doi:10.1007/s00299-020-02619-8)
Supplement: Supplementary file 6 — Supplementary file6 (DOCX 23 kb) [file 299_2020_2619_MOESM6_ESM.docx]

**Supplementary Method S1**

**Detailed Methodology**

**Isolation of genomic DNA**

DNA was isolated from leaf tissue by cationic hexadecyl trimethyl ammonium bromide (CTAB) method of Doyle and Doyle (1990)with minor modifications. . One gram of leaf tissue was frozen in liquid nitrogen and grounded to fine powder with a chilled mortar and pestle. Ten ml of extraction buffer [2% (w/v) CTAB (Sigma) 1.4 M NaCl, 0.1 M Tris-HCl (pH 8.0), 0.02 M EDTA (Sigma), 0.2% (v/v) 2- mercaptoethanol] was added to the grinded leaf tissue and the mixture was incubated at 60 ºC 45 min with occasional gentle swirling. It was then extracted twice with chloroform/isoamyl alcohol ( 24:1, v/v) and nucleic acid was precipitated with 0.7 volume of cold isopropanol , washed with 70% ethanol and the pellet was allowed to dry briefly at room temperature. Resuspended the pellet in nuclease free water. the DNA sample was then treated with RNAse A (final concentration 10 ug/ml) for 30 min at 37 ºC and diluted with 2 volumes of nuclease free water/TE. DNA was precipitated with 1/10^th^ volume of 3M sodium acetate and 2.5 volume of cold dehydrated ethanol. Subsequently DNA pellet was washed with 75% alcohol, air dried and resuspended in 10:0.1 TE..

**RNA extraction and first strand cDNA synthesis**

Total RNA from different samples were extracted using a Qiagen RNeasy plant kit according to the manufacturer’s instructions. The concentration and purity quotient of RNA were determined by the measurement of 260 nm absorbance and 260/280 nm absorbance respectively. The integrity of total RNA was determined by 2% agarose gel electrophoresis with ethidium bromide staining. The RNA was used as template for reverse transcription polymerase chain reaction (RT-PCR). cDNA synthesis was carried out according to the manufacturer’s instructions (Ambion, USA). The synthesized cDNA was stored at -80 ºC until further use.

**Isolation of *HbCAld5H* gene**

The gene specific primers for the PCR amplification of genes were designed using the whole genome sequence of *Hevea brasiliensis* RRII 105 clone (unpublished data). The total RNA was isolated from the 2-3^rd^ intermodal region of shoot using Pine tree method (Chang et al., 1993. The full length cDNA and genomic DNA fragment of *HbCAld5H* gene were amplified using cDNA template synthesized by RT-PCR (using primers CAld5HcD-F-5′AGGGAAGCCAGTGAATGTTG3′ and CAld5HcD-R-5′TCTATCCATCCAAGCCAAGG3′) and from genomic DNA (using primes CAld5H_GD-F-5′ CCATTACATCTTGCCCAACC3′ and CAld5H_GD-R-5′CCTGATTCGGTTTCATCGTC3′) respectively. The PCR reactions were performed with a denaturation temperature of 94 ºC for 4 min., then 35 cycles of 94 ºC for 30 s, 58 ºC for 45 s and 72 ºC for 1.5 min, with final extension for 7 min in an Eppendorf thermal cycler (Master cycler EP Gradient S, Germany). The PCR products were run on 1% agarose gel and the amplicon is purified using gel band purification kit (Qiagen, Germany). The purified DNA is then cloned into pGEM-T Easy Vector (Promega, USA) and transformed into *Escherichia coli* DH5α cell. The recombinant plasmids were identified and isolated from colony PCR positive clones and sequenced by the dideoxy-chain reaction termination method at SciGenome Laboratories Ltd, Cochin, India. The confirmed full length genomic DNA and cDNA of *HbCAld5h* was used for molecular characterization and bioinformatics analysis.

**Sequence comparison and phylogenetic analyses**

Both the nucleotide and amino acid sequences of *HbCAld5H* were analysed using NCBI blast program. The multiple sequence alignment of the *HbCAld5H* with other known plant CAld5H proteins were conducted using Clustal W program. The deduced amino acid sequence of *CAld5H* genes showing more than 70% identity with *HbCAld5H* were selected to construct a phylogenetic tree by MEGA 5.0 software (Saitou et al. 1987) using Neighbor Joining methods (Tamura et al. 2011) and the bootstrap test was carried out with 1,000 boot strap replicates. The number at each node represent the boot strap support (percentage).

**Development of sense and antisense binary vector constructs of *HbCAld5H***

For plant transformation experiments, cassettes with the sense and antisense constructs were made using an intermediate vector pRT 101 having 35SCaMV promoter at the upstream and NOS terminator at the downstream of the insert. To check the effect of length of antisense RNA on gene regulation, two constructs having antisense region of 842bp and 473 bp were prepared. These constructs were designated as coniferaldehyde-5-hydroxylase antisense (CAS) A (CAS-A) and B (CAS-B) respectively. Plasmid pGEM-T Easy vector containing *HbCAld5H* gene was digested with SacI, KpnI and BamH1 and SacI to release the antisense and sense fragments respectively. These fragments were subsequently cloned into linearized pRT101 vectors digested with the same restriction enzymes. The recombinant pRT101 vectors were digested with the restriction enzyme PstI to release the sense and antisense constructs along with CaMV35S promoter and NOS polyA terminator. These cassettes were cloned into pCAMBIA 1301 vector at the PstI site (Fig. S1). The recombinant binary vector was mobilized into *Agrobacterium tumefaciens* (LBA4404) for subsequent plant transformation experiments.

**Plant transformation and regeneration**

*Nicotiana tabacum* was transformed by pCAMBIA1301-sense and antisense *HbCAld5H1* constructs using the leaf-disk method. Untransformed tobacco plants served as controls. Hygromycin (50 mg/l) was used as a selective agent during *in vitro* regeneration. Differentiation of shoots was achieved on MS medium supplemented with 1 mg/l 6-benzylaminopurine (BA) and 0.1 mg/l napthaleneacetic acid (NAA). Rooting was obtained on MS basal medium devoid of growth regulators. Cefotaxime and carbenicillin were used at 250 mg/l each during *in vitro* regeneration to remove excess bacterial growth. Transformed plants were grown *in vitro* for 6 weeks under a light-dark regime of 16 h (20-30 mE m-2 s^-1^, 27 ^o^C)/8 h (27 ^o^C) and then transferred to soil and grown to maturity in the green house. T_0_ transformants were allowed to self-pollinate to obtain homozygous lines. T_1_ seeds were harvested and subjected to selection on germination medium containing hygromycin.

**Molecular characterization of transformants**

Genomic DNA was isolated according to Doyle and Doyle (1990), purified and quantified. PCR was performed with DNA isolated from transgenic and wild-type (WT) plants using *HbCAld5H* gene specific primers. The reaction volume for all PCR reactions was set to 25 µl. PCR amplifications were carried out starting with an initial denaturation at 94 ºC for 5 min, denaturation at 94 ºC for 30 s, annealing at 56 ºC for 45 s and extension at 72 ºC for 2 min. These steps were repeated for 35 cycles followed by a final extension for 5 min at 72 ºC. The reaction mixture without a template was run as a negative control. Positive controls for *HbCAld5H* were also included. Amplified DNA fragments were separated by gel electrophoresis in 1% agarose gel.

**Southern blot analysis**

Genomic DNA isolated from the leaves of transgenic tobacco and WT plants (10 µg each) was digested with SacI, separated by agarose gel electrophoresis and blotted onto nylon membranes (Hybond N, Amersham Biosciences, UK) using standard protocols (Sambrook and Russel 2001). DNA amplified with gene specific primers from pCAMBIA-sense and antisense *HbCAld5H* (CAS-A and CAS-B) plasmids were used as probes after labelling with DIG DNA labelling and detection system (Boehringer Mannheim). Both positive and negative controls were included. Hybridization was carried out overnight at 45 ºC in DIG easy HYB buffer (Roche). The blots were washed three times with 2X SSC, 0.1% SDS pH 7.0 for 5 min, then twice with 0.5X SSC, 0.1% SDS, pH 7.0 for 15 min at 45 ºC. Blots were washed once with the buffer containing 0.1 M maleic acid, 0.015 M NaCl, pH 7.5. The visualization of the probe-target hybrid was achieved by a chemiluminiscent assay using the DIG Luminiscent Detection Kit protocol (Roche).

**Real time PCR analysis**

Total cDNA was used as the template for q-RT-PCR. The first-strand cDNA was prepared from leaf tissues of WT and transgenic tobacco plants according to the manufacturer’s protocol of Ambion cDNA Kit (Thermo Scientific, USA) and stored at -80 ºC. For gene expression study in *Hevea*, cDNA was synthesized from the total RNA isolated from shoot tip, internodes 2-3 (beginning of active xylogenesis) internode 4-5 (with complete ring of secondary xylem) and root tip of 1 year old plant and also from the main trunk of 20 year old tree. The tissue specific expression of *HbCAld5H* was determined by qRT-PCR using gene specific primers. The efficiency and specificity of primers was detected before performing the qRT-PCR analysis process. The qRT-PCR results were measured by using 7500 SDS software (Applied Biosystems, USA). Tobacco β-actin, considered to be one of the most suitable reference genes in terms of stability for real-time PCR experiments (Bustin 2002; Schmidt and Delaney 2010) has been used as a reliable reference gene to find out the expression levels of *HbCAld5H* gene in transgenic tobacco (Chen et al. 2019). Therefore, the expression levels of tobacco β-actin gene as the reference gene with actin specific primers were quantified in parallel with the target genes as the internal control. Three biological samples and triplicate technical qRT-PCR reactions for each combination of primers and samples were analysed.

**Light microscopy**

For general histology, transverse sections (1-2 µm thick) taken with a diamond knife from the LR White embedded samples using an ultra-microtome (Leica UMO7, Germany) were stained with 0.05% toluidine blue O (Berlyn and Mikshe 1976). Sections were examined and photographed using Leica microscope (DM200) with a Canon digital camera (DM 150). For histochemical analysis, hand sections taken from stem and leaf tissues of control and transgenic lines were stained with toluidine blue O for general histology, phloroglucinol-HCl for lignin localization (Speer 1987) and Maule’s reaction (Meshitsuka and Nakano 1979) for syringyl lignin localization. Stained sections were observed and photographed using Leica DM200 microscope (Germany). Stem samples from control, lignin up- and down-regulated plants were macerated to measure the length and width of fibres and vessel elements. Small matchstick size stem pieces were macerated by incubating in Jeffrey’s fluid (Berlyn and Miksche 1976).After thorough washing in water, the macerated elements were stained with safranin O (Sigma, S-2255) before mounting in 50% glycerol. The length and width of fibres and vessel elements were measured with an ocular micrometer scale mounted in a research microscope. For each parameter, 100 readings were taken from randomly selected elements and they were statistically analyzed to determine the mean values.

**Transmission electron microscopy**

Suitably trimmed (2×5 mm size) pieces of stem tissues were fixed in a mixture of 0.1% glutaraldehyde and 4% paraformaldehyde in 50 mM sodium cacodylate buffer for 4 h at room temperature. After washing in buffer, tissues were dehydrated in graded series of ethanol (30-95%, 15 min each, pure ethanol × 3, each for 20 min) and embedded in LR white resin as described elsewhere (Pramod et al. 2019).

**Lignin localization**

Transverse ultrathin sections having 70-80 nm thickness were prepared from the LR white embedded blocks using an ultra-microtome (Ultracut E, Leica, Germany) with a diamond knife and mounted on nickel grids. Sections were stained with 0.1% KMnO_4_ in citrate buffer for 45 minutes at room temperatures for lignin (Donaldson 1992).

**Immunogold labelling**

Ultrathin sections of 90 nm thickness were taken from the LR white embedded blocks were mounted on nickel grids. After suspension of the grids in buffer ‘A’ (composition: Tris-buffered saline containing 1% bovine serum albumin and 0.1% NaN_3_, pH 8.2) for 30 min at room temperature, the grids were incubated with LM5, CCRCM1, LM10 or LM11 antibodies (1:20 dilution in buffer A) for 2 days at 4 ˚C. The labelling method was same for xyloglucan except the grids were incubated with goat antimouse secondary antibody labelled with 5 nm colloidal gold particle (BB International, UK). After three washings with buffer A for 15 min each, the grids were incubated with goat anti-rat secondary antibody labelled with 10 nm colloidal gold particles (BB International, UK) for 2 h at room temperature for the LM10 antibody (1:20 dilution in buffer ‘A’). For the control, some sections were also incubated only with secondary antibody. Finally, the grids were washed in six changes of buffer ‘A’ for 15 min each, followed by washing with distilled water. Ultrathin sections were post-stained with 1% KMnO_4_ for 30 min, washed in three changes of distilled water for 10 min each. All sections were examined under a transmission electron microscope (TEM, JEOL 1420) at an accelerating voltage of 120kV.

**Lignin analysis**

Lignin content analysis was carried out on dry extractive-free cell wall residue from stem which was ground to pass through a 180 µm sieve before exhaustive solvent extraction [2:1 (v/v) toluene:ethanol, ethanol and water]. The lignin content in the control and transgenic lines was determined by the Klason method (Dence 1992). For determination of S/G ratio, thioacidolysis was carried out according to Lapierre et al. (1995). The reagent was prepared by introducing 2.5 ml of BF3 etherate (Aldrich) and 10 ml of ethanethiol (Aldrich) into a 100 ml flask and the final volume adjusted to 100 ml with dioxane. A mixture of the sample (12 mg) and 12 ml of reagent were put in a tube fitted with Teflon lined screw cap. Thioacidolysis was performed at 100 ˚C (Oil bath) for 4 h with an occasional shaking. The cooled reaction mixture and the washings with water were combined with and the mixture was poured over 1 ml dichloromethane (Fluka, Germany) including internal standard (0.50 mg tetracosane from Sigma, Germany). After adjusting the pH of the aqueous phase to pH 3-4 with 0.4 M sodium carbonate aqueous solution, the aqueous phase was then extracted with dichloromethane (20 ml×3). The combined organic extracts were dried over Na_2_SO_4_ and the solvent was evaporated under reduced pressure at 40 ˚C in a rotary evaporator. The residue was redissolved in dichloromethane (1 ml). The thioacidolysis products (7 µl) were sylylated with 50 µl of N, O-bis (trimethylsilyl) trifluroacetamide (BSTFA, Sigma, Germany) and 5 µl of pyridine (Sigma, Germany) in a 200 µl GC vial with Teflon lined screw cap and kept at room temperature for overnight. The sylylated products were separated by Gas chromatography using silicon based capillary column (30 m×0.25 µm×250 µm) and each peak was identified by GC/MS. The temperature program of the GC increased at a rate of 5 ˚C/min from 100 ˚C/280 ˚C and then final temperature was maintained for 60 min.

**Statistical analysis**

Student t-test was carried out to determine statistically significant differences of anatomical and biochemical parameters at a 0.05 confidence level using Sigmastat software (Version 3.5, San Jose, CA, USA).

**CAld5H cDNA complete sequence mRNA (1542bp): Antisense construct positions**

CAS A: Base numbers in CDS 696 to 1538 (842bp)

CAS B: Base numbers CDS 156 to 628 (473 bp)

**ATG**GAAGCTCTTCTCCAAGCTCTGCAACCTTTACCCTTCACTCTTTTCCTTATTGTTCCTTTACTATTCCTACTAGGTCTAATCTCTCTCCTTCGTAGACGATTGCGATATCCTCCAGGGCCTAAAGGCTTGCCAATTATAGGTAACCTGCTCATGATGGACCAGTTAACTCACCGGGGTTTAGCTAAACTAGCTAAAGAATATGGTGGCCTCTTCCATCTTCGCATGGGAAACATTCACATGTTTGCTGTTTCTTCTCCTGAGATAGCTCGCCAAGTACTTCAAGTCCAAGATAATATTTTCTCAAATAGACCTGCCACCATAGCCATCAGTTATCTCACATATGACCGTGCAGATATGGCCTTCGCCCACTACGGCCCTTTCTGGCGCCAGATGCGCAAGCTCTGCGTCATGAAACTTTTCAGCCGTAAACGGGCTGAGTCATGGGAATCTGTGAGGGATGAGGTTGATTATATGGTTAAAACCGTTGTGGCCAACAAAGGGAAGCCAGTGAATGTTGGAGAGTTGATTTTTACTCTAACCATGAATATTATTTATAGGGCTGCTTTCGGTTCGAAGAACGAGGGACAGGACGAGTTTATTCAGATTTTGCAGGAGTTCTCCAAGCTTTTTGGTGCTTTCAACATTGCTGATTTCATTCCTTGGCTTGGATGGATAGACCCACAAGGCCTCAAGCTCAACTCAAGACTTGCCAAGGCTCGTAAATCACTTGACAGATTCATCGACTTGATCATCGACGAACACATGCATAAGAGTAAGCAGGGTAATGTCTCTGATGATAACTCTGATATGGTGGATGATTTGCTAGCTTTCTACAGCGATGAGCCTAAAGTAAACGAATCAGACGATCTTCAGAATTCCTTCAAACTCACTCGAGACAACATCAAAGCAATTATCATGGACGTAATGTTTGGTGGGACAGAGACAGTGGCATCGGCGATTGAGTGGGCCCTGACGGAGTTGATGAGGGCTCCTGAAGAGTTGAAAAAGGTCCAGCAAGAGCTTGCCGATGTGGTGGGTCTAGAGCGGCGCGTGGAGGAGAGTGATTTCGACAAGCTGACATACCTAAAATGCACAGTCAAAGAAACTCTAAGACTTCACCCACCAATCCCACTTCTCTTACATGAGACGGCCGAGGAGGCAGAGGTCGCCGGGTATTACATTCCAGCGAAGTCTCGGGTCATGATCAATGCATGGGCTATTGGAAGGGACAAGAACTCATGGGAGGACCCTGAAACTTTCAGGCCATCCAGGTTTTTGAAAGAGGGAGTGCCTGATTTTAAAGGAAACAATTTCGAGTTCATTCCATTCGGATCGGGCCGGAGATCCTGCCCCGGCATGCAACTCGGACTCTACTCTCTTGATTTGGCTGTGGCCCATCTTCTTCACTGTTTTACATGGCAGTTGCCTGATGGGATGAAACCAAGTGAAATTGACACGAGCGATGTGTTTGGACTCACCGCTCCTCGGGCAACCCGACTGGTAGCTGTTCCAAACGCGCGTTTGTTGTGCCCGCTC**TAA**
